# Porous Laser-Scribed Graphene Electrodes Modified with Zwitterionic Moieties: A Strategy for Antibiofouling and Low-Impedance Interfaces

**DOI:** 10.1021/acsami.3c15849

**Published:** 2024-01-17

**Authors:** Alanis C. Zambrano, Livia M. D. Loiola, Abdullah Bukhamsin, Radoslaw Gorecki, George Harrison, Veerappan Mani, Shadi Fatayer, Suzana P. Nunes, Khaled N. Salama

**Affiliations:** † Bioengineering Program, Biological and Environmental Sciences and Engineering Division, King Abdullah University of Science and Technology (KAUST), 23955-6900 Thuwal, Saudi Arabia; ‡ Advanced Membranes and Porous Materials Center, King Abdullah University of Science and Technology (KAUST), 23955-6900 Thuwal, Saudi Arabia; § Environmental Science and Engineering Program, Biological and Environmental Sciences and Engineering Division, King Abdullah University of Science and Technology (KAUST), 23955-6900 Thuwal, Saudi Arabia; ∥ KAUST Solar Center, King Abdullah University of Science and Technology (KAUST), 23955-6900 Thuwal, Saudi Arabia; ⊥ Applied Physics Program, Physical Science and Engineering Division, King Abdullah University of Science and Technology (KAUST), 23955-6900 Thuwal, Saudi Arabia; # Chemistry and Chemical Engineering Programs, Physical Science and Engineering Division, King Abdullah University of Science and Technology (KAUST), 23955-6900 Thuwal, Saudi Arabia; ¶ Computer, Electrical and Mathematical Sciences and Engineering Division, King Abdullah University of Science and Technology (KAUST), 23955-6900 Thuwal, Saudi Arabia

**Keywords:** laser-scribed graphene, zwitterionic, antibiofouling, biosensors, point-of-care diagnostics

## Abstract

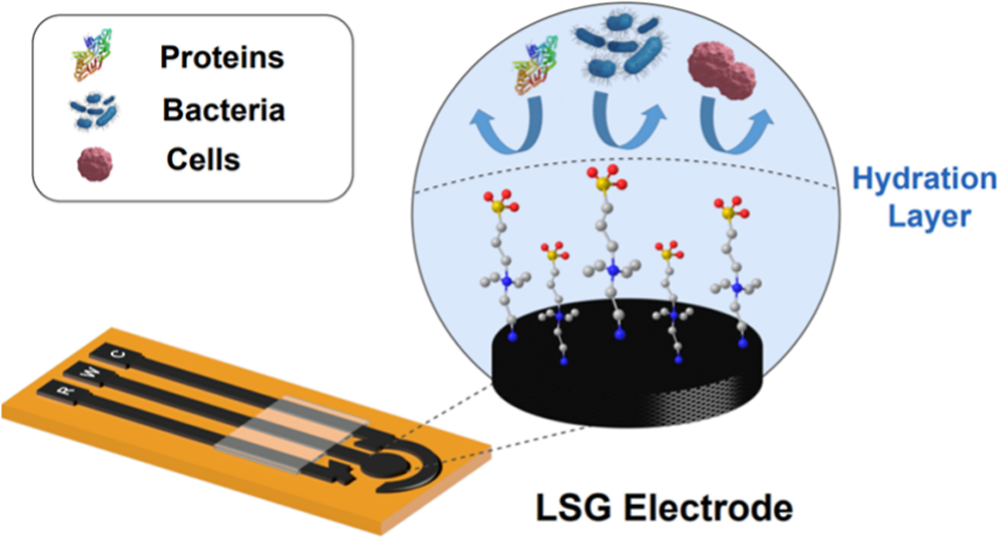

Laser-scribed graphene electrodes (LSGEs) are promising platforms for the development of electrochemical biosensors for point-of-care settings and continuous monitoring and wearable applications. However, the frequent occurrence of biofouling drastically reduces the sensitivity and selectivity of these devices, hampering their sensing performance. Herein, we describe a versatile, low-impedance, and robust antibiofouling interface based on sulfobetaine-zwitterionic moieties. The interface induces the formation of a hydration layer and exerts electrostatic repulsion, protecting the electrode surface from the nonspecific adsorption of various biofouling agents. We demonstrate through electrochemical and microscopy techniques that the modified electrode exhibits outstanding antifouling properties, preserving more than 90% of the original signal after 24 h of exposure to bovine serum albumin protein, HeLa cells, and *Escherichia coli* bacteria. The promising performance of this antifouling strategy suggests that it is a viable option for prolonging the lifetime of LSGEs-based sensors when operating on complex biological systems.

## Introduction

During the past decade, graphene-based materials have become a promising platform for the development of electrochemical biosensors.^1,2^ Recently, laser-scribed graphene electrodes (LSGEs) have demonstrated enhanced analytical performance due to their unique chemical and physical properties.^3−5^ Owing to their dense distribution of edge defect sites, the high surface potential energy of LSGEs can support a rapid rate of electron transfer. Combined with their three-dimensional (3D) porous structure, LSGEs retain a large specific surface area and suitable electrical conductivity.^4,6,7^ Furthermore, LSGEs can be manufactured by an easy and cost-effective method via low-power laser ablation of a carbon-containing precursor without any complex mask patterning.^8^ This allows for the mass production of flexible, miniaturized, and customizable LSGE-based devices.^2,9^ For instance, LSGE and their nanocomposites have been applied successfully in high-performance flexible glucose sensors.^10,11^ Moreover, LSGEs are known for their resilience to the typical mechanical deformations experienced by wearable devices such as bending, twisting, or stretching.^12,13^ All these characteristics and advantages have been exploited for the development of electrochemical biosensors for several applications, including the fabrication of functional circuits, in situ continuous monitoring of human biomarkers, real-time drug monitoring, environmental monitoring of chemical contaminants and pesticides, food safety, and wearable sensors.^14−20^

These devices are routinely exposed to complex biological fluids such as blood, serum, saliva, urine, and sweat.^21,22^ This makes them susceptible to the rapid loss of selectivity and sensitivity caused by biofouling.^23−25^ Biofouling results from the unwanted adsorption of biological molecules on surfaces, which include proteins, bacteria, cells, lipids, and polysaccharides.^24−26^ At the electrode surface, it usually involves the formation of an impermeable passivating layer driven by nonspecific interactions, depending on the type of biofoulant and the electrode material.^26^ Biofouling is often regarded as the main cause of contamination of biosensing devices and operational failure, which reduces their reliability. It severely impairs the electrochemical performance by increasing background noise. Moreover, biosensors cannot yet be deployed in vivo as their biofouling can lead to inflammation, encapsulation of the tissue that surrounds the electrode, infections, and thrombosis.^27−29^

Numerous antifouling strategies have emerged to address this challenge.^30^ The most thoroughly investigated strategy centers on the modification of the surface chemistry by incorporating materials with antifouling properties.^31^ Ethylene glycol (EG) and zwitterionic (ZW)-based materials are the preferred choice and have been widely used in biomedical applications.^32−35^ Both possess a hydrophilic nature and are capable of forming a strong interfacial hydration layer that repels biofouling agents.^33^ However, recent studies have shown that EG-based coatings can undergo oxidative degradation and elicit an immunogenic response.^36^ On top of that, the functionalization of these polymers on the electrode surface reduces the electrochemically active surface area and slows down the electron-transfer process due to their insulating nature.^25,37^ In contrast, electroneutral ZWs possess an enhanced water-holding capacity via ionic solvation, thereby making the hydration layer robust.^38^ Additionally, ZWs have superior hemocompatibility, which reduces the risk of infection and thrombosis.^30,39^ Consequently, ZW-based materials have been broadly applied in different platforms such as nanoparticles, nanofibers, membranes, and biosensors.^36,40−42^ Many types of ZWs have been investigated, such as carboxybetaine, phosphobetaine, and sulfobetaine derivatives. Among those, sulfobetaine derivatives emerged as the most promising candidates due to their economical and simpler synthesis.^31^ In addition, sulfobetaine groups are known for their great thermal stability, chemical stability, and pH stability.^43,44^ Unlike poly(ethylene glycol) (PEG)-based materials, sulfobetaine ZWs are less likely to trigger an immunogenic response due to their ability to enhance the hemocompatibility of different materials and their antibiofouling capacity.^36,45^ For instance, sulfobetaines used in delivery systems exhibited negligible cytotoxicity and insignificant immune response.^46^ To the best of our knowledge, the combination of ZWs with LSGEs has not been explored to date.

In this study, working electrode surfaces of LSGEs were functionalized with sulfobetaine-ZW moieties to provide an antifouling interface with low impedance, which maintains a high electrochemical response after exposure to various biofouling agents. On the basis of maximizing the antifouling effect while maintaining the sensitivity of the LSGEs, the density of the ZW interface at the electrode surface was optimized. The modified electrodes were electrochemically characterized by differential pulse voltammetry, cyclic voltammetry, and electrochemical impedance spectroscopy using an electrochemically active solution of potassium ferri-ferrocyanide. We demonstrate that the ZW-modified LSGEs exhibit excellent antifouling properties against bovine serum albumin (BSA), HeLa cells, and *Escherichia coli* bacteria even after 24 h of incubation. Our findings indicate that over 90% of the electrochemical current response is preserved in the modified electrodes after exposure to biofoulants. This stands in stark contrast to bare LSGEs, which lose around 46, 32, and 14% of their original current after protein, cell, and bacterial adhesion, respectively.

## Materials and Methods

### Production of LSGEs

A 150 W CO_2_ laser (Universal Laser Systems PLS6.150D, λ = 9300 nm) was used to selectively graphitize a carbon-rich substrate via thermally driven rearrangement. An insulating Kapton polyimide layer (Utech Products; thickness = 200 μm) was used as the substrate. The optimal laser scribing speed was found to be 4.5 cm/s with 4.2 W of power. Resistance values that vary between (170 to 220 Ω) were obtained for each LSGE. The laser line density, definition, and contrast were set to 70.0, 15.0, and 18.0%, respectively. Furthermore, a sharp black-and-white dithering pattern was used during the scribing process. The structural features of the LSGEs including the presence of defects were analyzed by using Raman spectroscopy (λ = 532 nm, Horiba LabRAM Aramis).

### Sulfobetaine-ZW Synthesis

3-[(2-Aminoethyl) diethylammonium] propane-1-sulfonate was synthesized from the reaction between *N*,*N*-diethylethylenediamine (Sigma-Aldrich) and 1,3-propane sultone (Sigma-Aldrich), as reported previously.^47^ Diethylethylenediamine (43 mmol) and propane sultone (60 mmol) were dissolved in 5 mL of dry acetonitrile, and the reaction medium was stirred for 24 h under an argon atmosphere with the temperature set to 50 °C using an oil bath. Afterward, the product was centrifuged at 5000 rpm for 5 min and washed with fresh dry acetonitrile five times. Later, the purified sample was frozen in liquid nitrogen and dried under vacuum for 48 h using a Labconco freeze dryer. Attenuated total reflectance Fourier transform infrared spectroscopy (ATR-FTIR) measurements of the ZW compound were performed on a Thermo Scientific Nicolet iS10 FTIR spectrometer at room temperature by recording 32 accumulated scans at a resolution of 4 cm^–1^ in the 4000–600 cm^–1^ wavenumber range. Proton nuclear magnetic resonance (^1^H NMR) spectrum of ZW compound in deuterium oxide was acquired on a Bruker AVANCE 400 MHz spectrometer using a 5 mm gradient probe by recording 64 scans. Data analysis was performed using Bruker TopSpin 4.0 software, and the calibration was based on the residual water signal at 4.80 ppm. Additionally, accurate mass determination of the ZW compound was acquired in positive ion mode using a Bruker compact quadrupole time-of-flight (QTOF) mass spectrometer equipped with a heated electrospray ionization (ESI) ion source. A 0.01 mg/mL compound solution in HPLC-grade methanol was infused inside the ESI chamber at a 100 μL/min sample rate using a Fisher Scientific Syringe pump. Sodium formate clusters were used for mass calibration, and the analysis was performed under the following conditions: mass range from 50 to 1000 *m*/*z*; end plate offset of 500 V; capillary voltage of 4500 V; source temperature of 200 °C; nebulizing gas pressure of 0.8 bar; and drying gas flow of 4.5 L/min. Data analysis was performed using Bruker Compass software.

### ZW Anchoring on LSGE

The surface of the LSGEs was cleaned with DI water and dried with a gentle stream of nitrogen prior to use. Carbodiimide chemistry was applied to covalently bind the sulfobetaine ZW to the working electrode surface. The coupling agents, ethyl(dimethylamino propyl)carbodiimide (Sigma-Aldrich) and *N*-hydroxy sulfosuccinimide (Sigma-Aldrich) were dissolved in a 10 mM 2-(*N*-morpholino) ethanesulfonic acid buffer (Sigma-Aldrich, pH 4.50), leading to a final coupling agent solution with 0.25 M of carbodiimide and 0.25 M of sulfosuccinimide. Each working electrode was immersed in 3.0 μL of a coupling agent solution for 30 min. Immediately after, the coupling agent solution was removed, and 3.0 μL of a 2.0 M ZW solution in the pH 4.50 buffer was immersed for another 30 min to ensure the completion of the reaction.^48^ Lastly, the surface was washed three times by immersing the electrode in a copious amount of DI water for 5 min to remove excess organisms.

For comparative studies, LSGEs were also coated with PEG by reacting poly(ethylene glycol)methyl ether amine (*M*_n_ = 500, Sigma-Aldrich) instead of ZW, after LSGE treatment with the coupling agent.

### XPS Analysis

X-ray photoelectron spectroscopy (XPS) was performed on bare LSGEs, the control LSGE/coupling agent, and LSGE/ZW to confirm the functionalization of the ZW moieties onto the surface of the electrodes. XPS was performed in a UHV system (Scienta Omicron) that utilizes an Argus CU analyzer and a XM1000 monochromatic X-ray source under charge neutralization. XPS spectra were calibrated by reference to the C–C/C–H peak at ca. 285 eV.^49^

### Electrochemical Characterization

All experiments were carried out using LSGEs containing a working electrode with a geometric area of 3 mm^2^. The working electrode was either LSGE/ZW or unmodified LSGE. The counter and reference electrodes were LSGE. The electrodes are connected to a potentiostat (PalmSens 4, PalmSens BV) through a screen-printed electrode connector (4 mm banana cable). The antifouling coatings were electrochemically characterized using a redox aqueous solution of potassium ferri-ferrocyanide [5.0 mM K_4_Fe(CN)_6_/K_3_Fe(CN)_6_ in 0.1 M KCl] by differential pulse voltammetry (scan rate 0.1 V s^–1^ between −0.4 and 0.5 V), cyclic voltammetry (scan rate 0.1 V s^–1^ between −0.5 and 0.5 V), and electrical impedance spectroscopy (0.1 Hz to 100 kHz, 5 mV sinusoidal excitation voltage overlaid on a DC offset of +0.10 V, 70 frequencies logarithmically spaced, 9 frequencies per decade), before and 24 h after immersion in the albumin solution.

### Protein, Cell, and Bacterial Adsorption Assays

Nonspecific adsorption of proteins onto the LSGE surface was assessed using 10, 30, and 50 mg mL^–1^ bovine albumin (Sigma-Aldrich) diluted in 0.01 mmol/L phosphate buffer (Fisher BioReagents, pH = 7.4). The surface of the working electrode of the LSGEs was immersed for 24 h in 3.0 μL of the albumin solution and kept in a closed water-saturated environment to avoid water evaporation. The adsorption of this organic fouling agent was evaluated electrochemically by using differential pulse voltammetry, cyclic voltammetry, and electrochemical impedance spectroscopy and investigated by scanning electron microscopy (SEM) and fluorescence microscopy. In addition, myoglobin from equine skeletal muscle (Sigma-Aldrich) and lysozyme (Sigma-Aldrich) in a concentration of 30 mg mL^–1^ were tested and evaluated using differential pulse voltammetry to investigate their fouling effect on LSGE and LSGE/ZW.

Additionally, the biofouling effect caused by cellular adhesion was carried out using HeLa cells (T-REx Cell Line, Thermo Fisher Scientific). The cells were cultured for 48 h into a cell plate at 37 °C and 5% CO_2_ with the medium being changed every 24 h. The medium consists of Dulbecco’s modified Eagle medium, fetal bovine serum, and penicillin–streptomycin in a ratio of (10:1:0.1), respectively. The cells were stained using trypan blue and counted (2.58 × 10^6^ cells/mL) using an automated cell counter (Countess 3 FL). A 3.0 μL portion of the cells in the media was directly incubated on top of the electrodes to conduct the fouling test.

Similarly, the adhesion and growth of bacteria to the working electrode were tested by using *E. coli* (*E. coli*, DSM 1103). *E. coli* was cultured using a Luria–Bertani agar plate at 37 °C for 24 h. Subsequently, one single colony was picked and cultured overnight using a shaking incubator (37 °C, 220 rpm) in Luria–Bertani broth liquid media. The colony-forming unit (approximately 3 × 10^9^ cfu/mL) and optical density (OD_600_ = 1.68) were measured from the stock bacterial suspension. The *E. coli* suspension used in this experiment was diluted with 0.01 mol/L phosphate-buffered saline (Thermo Fisher Scientific, pH = 7.40) to obtain a concentration of approximately 8 × 10^7^ cfu/mL. A 3.0 μL portion of the diluted *E. coli* suspension was directly incubated on top of the electrodes to conduct the fouling test.

The electrochemical studies of the electrodes upon incubation with HeLa cells and *E. coli* were performed using differential pulse voltammetry, using the same protocol and parameters as those for albumin solutions.

### Morphological Characterization by SEM

The analysis was carried out on bare and modified electrodes before and after immersion in a 10 mg mL^–1^ albumin solution to inspect the surface topography and morphology. The samples were rinsed with DI water three times to wash out unattached species. All the samples were mounted on the SEM stubs with the use of double-sided adhesive copper conductive tape (3 M Company, MN, USA) and sputter-coated with 5 nm of gold using a turbomolecular pumped coater Q300TD (Quorum Technologies, UK). The SEM analysis was conducted using Quattro ESEM (Thermo Fisher-FEI), with an accelerating voltage of 5 kV, a current of 28 pA, and a working distance of 10 mm.

### Fluorescence Imaging Analysis

The adsorption of proteins to the surface of the electrodes was investigated by comparing the fluorescence intensities of bare LSGE and LSGE/ZW incubated with albumin. For this, albumin-fluorescein isothiocyanate conjugate (FITC-albumin, Sigma-Aldrich) was dissolved in 10X phosphate-buffered saline (pH 7.40) to a 10 mg mL^–1^ concentration. The working electrode was immersed into the fluorescein-conjugated albumin solution for 24 h and gently rinsed afterward with DI water, to wash out unattached species. A stereo microscope (Nikon SMZ25) was used to acquire the magnified fluorescent image of the working electrodes. The intensity of fluorescence was analyzed via image segmentation by isolating the green channel’s average intensity in a pixel-by-pixel procedure.

### Contact Angle Measurements

The hydrophilicity of bare LSGE and modified LSGE/ZW surfaces were evaluated by contact angle measurements using a DSA100E droplet shape analyzer (Kruss, Germany). For this, a 1.0 μL droplet of water was applied on the working electrode surface and photographed using a digital camera. Image acquisition and droplet shape analysis were performed by using ADVANCE software. It is important to point out that all samples were freeze-dried overnight prior to contact angle measurements.

### Shelf Life of the LSGE/ZW

The stability of the LSGE/ZW-modified electrodes was assessed over a period of 4 weeks. For this, the electrodes were stored at 4 °C in Petri dishes and sealed with parafilm to insulate the samples from humidity and environmental contaminants. After immersion for 24 h in a 10 mg mL^–1^ albumin solution, differential pulse voltammetry was used to evaluate the viability of the modified electrodes every week.

## Results and Discussion

### Synthesis and Characterization

**Scheme 1:**
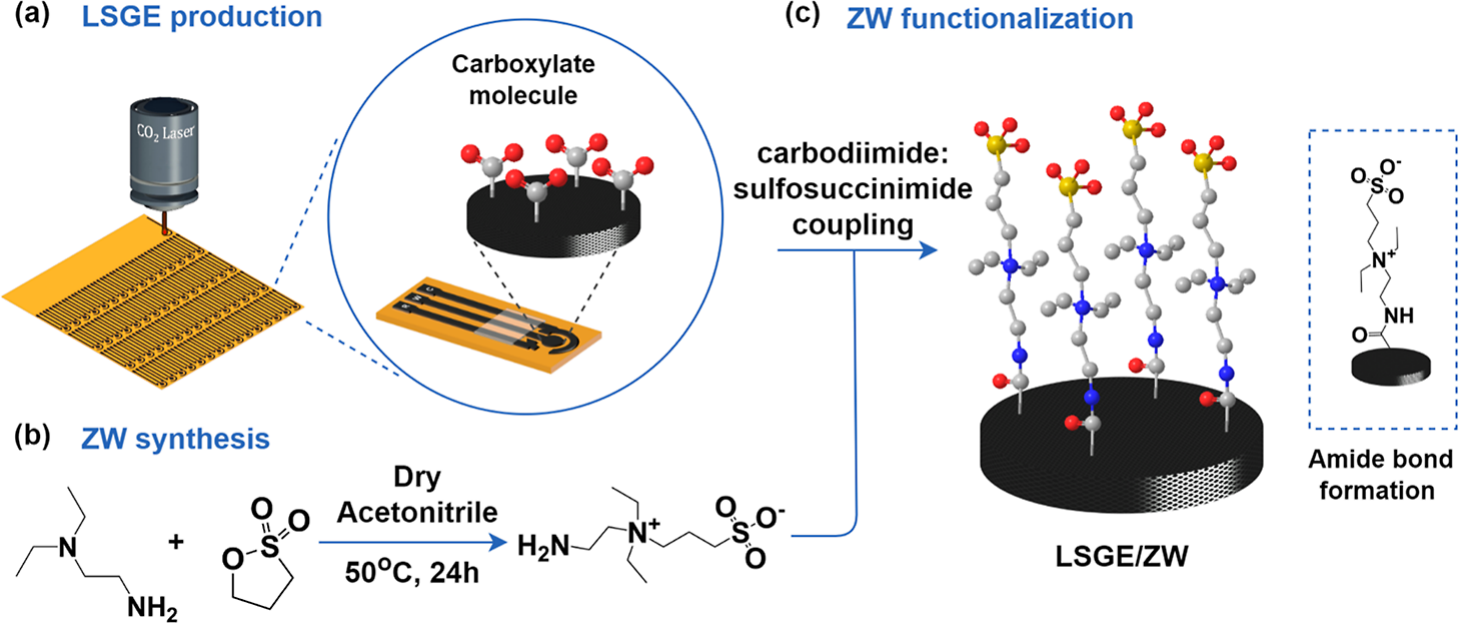
(a) LSGE Fabrication via CO_2_ Laser Irradiation; (b) ZW Synthesis; (c) ZW Functionalization of the Working Electrode via Carbodiimide Chemistry

Highly conductive, porous, and flexible electrodes were fabricated by using the laser-scribing technique (Scheme 1a). Briefly, multilayers of graphene are formed on a polyimide substrate by a CO_2_ laser irradiation. The scribed pattern was dictated via computer numerical control (CorelDraw and Universal Control Panel, ULS). This technique allows the electrical properties of the material to be modified by manipulating the intensity of the laser.^6^ In this study, we used a laser power of 2.8% to fully graphitize the substrate and produce highly conductive electrodes. The resistance of each LSGE was measured from pole to pole, obtaining values in the range of 170 to 220 Ω. Each device is composed of a three-electrode system containing a reference electrode, a working electrode, and a counter electrode. As the working electrode interface is crucial for analyte oxidation and reduction, any obstruction of the interface can impair performance. Here, we modified the working electrode of the LSGEs with ZW moieties to exhibit fouling resistance. The ZW compound was synthesized through the 1,3-propane sultone ring-opening reaction by the tertiary amine group of *N*,*N*-diethylethylenediamine, as illustrated in Scheme 1b. The resulting compound is yellowish, solid, and highly soluble in water. The structure and chemical properties of the material were characterized by FTIR, ^1^H NMR, and mass spectrometry (Figure S1a–c).

The ZW moieties were functionalized onto the working electrode surface through carbodiimide chemistry (Scheme 1c). The carbodiimide/sulfosuccinimide coupling agents first react with the carboxyl groups (−COOH) on the LSGE surface and then anchor the ZW functionality by reacting with the primary amine group of the sulfobetaine ZW, forming amide bonds.

**Figure 1:**
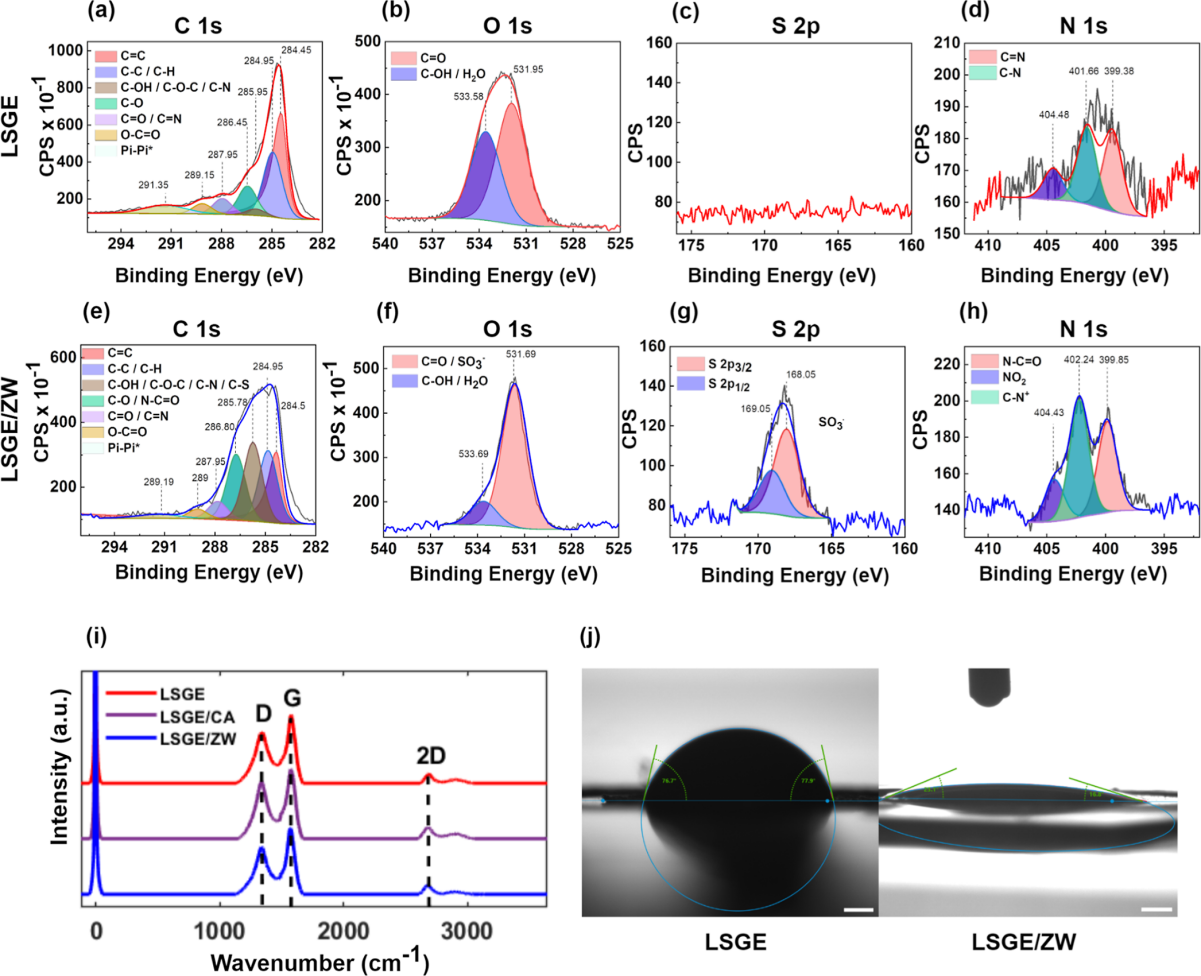
(a–d) XPS C 1s, O 1s, S 2p, and N 1s deconvoluted spectra of bare LSGE and (e–h) LSGE/ZW. (i) Raman spectra of bare LSGE (red line), LSGE/CA (purple line), and LSGE/ZW (blue line). CA: carbodiimide/sulfosuccinimide coupling agent. (j) Contact angle measurements using a water droplet on the working electrode surface of bare LSGE and LSGE/ZW (scale bar = 1 mm).

The XPS spectra of the bare LSGE and modified LSGE/ZW were compared to ensure the appropriate ZW functionalization of the working electrode surface. First, the spectra of C 1s, O 1s, S 2p, and N 1s were analyzed in bare LSGE. In Figure 1a, the deconvolution of the C 1s spectra showed six peaks, corresponding to C=C, C–C/C–H, C–OH/C–O–C/C–N, C–O, C=O/C=N, O–C=O bonds, and one peak at 291.35 eV from the contribution of a π–π* transition.^49^ The presence of the satellite peak indicates the aromaticity of the carbon present in the sample.^50^ The presence of the peaks at 289.15, 287.95, 286.45, and 285.95 eV suggests that the surface of the LSGE contains a mixture of –COOH, –C=O, and –C–OH functional groups. There is a small nitrogen contribution, which could be attributed to the polyimide substrate used in the production process, which is evident in the N 1s spectrum at ∼401.6 and ∼399.4 eV (Figure 1d). The O 1s spectrum revealed two peaks that confirm the presence of C=O and C–OH functional groups on the surface at 531.95 and 533.58 eV, respectively (Figure 1b). As depicted in Figure 1c, no evident S 2p peaks were observed for the bare LSGE.

The C 1s spectrum of modified LSGE/ZW electrodes revealed a substantial increase of 68.2% in the relative atomic percentage of N–C=O attributed to the formation of amide bonds (Figure 1e). Additionally, an increase in the XPS intensities of O 1s and S 2p is observed due to the contribution of the ZW terminal group (SO_3_^–^) (Figure 1f,g). An increase in the N 1s intensity was evident and coincided with two distinguishable peaks at 399.85 and 402.25 eV, corresponding to the formation of the amide bond and the N^+^ presence in the ZW moiety structure (Figure 1h).

Additional XPS analysis of LSGE modified with the carbodiimide/sulfosuccinimide coupling agent, which is an intermediate before anchoring the ZW functionality, was performed (Figures S2 and S3). An overall reduction in the C=C graphene peaks and –OH environment, as well as a slight increase in the S and N species due to the binding of the coupling agents was observed.

After confirming the appropriate binding of the ZW moieties onto the working electrode surface, Raman spectroscopy was performed to analyze the structural features of bare LSGE, LSGE/carbodiimide/sulfosuccinimide, and modified LSGE/ZW (Figure 1i). In the spectra, three characteristic bands of graphene are observed. The significantly pronounced D-band (∼1350 cm^–1^) confirms the abundance of defects in the samples, which is correlated with its inherent large surface area. The G-band (∼at 1580 cm^–1^) indicates the presence of sp^2^ hybridized planar carbon and demonstrates the stacking of graphene multilayers. The intensity ratio of the D and G bands (*I*_D_/*I*_G_) was calculated to monitor any changes in the degree of disorder or graphene defects after functionalization with ZWs. For this, different areas of each electrode surface were analyzed, and a normal distribution was assumed. The results showed no significant differences in the *I*_D_/*I*_G_ ratio between LSGE and LSGE/ZW, inferring that the functionalization caused no alterations in the degree of defects in the graphene structure inherent of LSGE. Finally, the 2D-band (∼2685 cm^–1^) showed no significant difference between bare LSGE and modified LSGE/ZW. This implies that there was no variation in the thickness of the graphene multilayer.

The chemical modification of the LSGEs’ surface with ZW moieties aims to increase the overall working electrode hydrophilicity to provide the antibiofouling effect, as it has been demonstrated that interfacial dehydration facilitates the irreversible adhesion of biological media.^33^ To demonstrate this, a wettability test was performed by measuring the contact angle of a water droplet on a bare LSGE and a modified LSGE/ZW (Figure 1j). As expected, the bare LSGE surface presents a contact angle with the water drop at around 80°, while LSGE/ZW surfaces are immediately and completely wet after the water drop addition, which reflects the increase of the overall surface hydrophilicity after the ZW moieties bonding.

### Electrochemical Assessment of the ZW Interface

**Figure 2:**
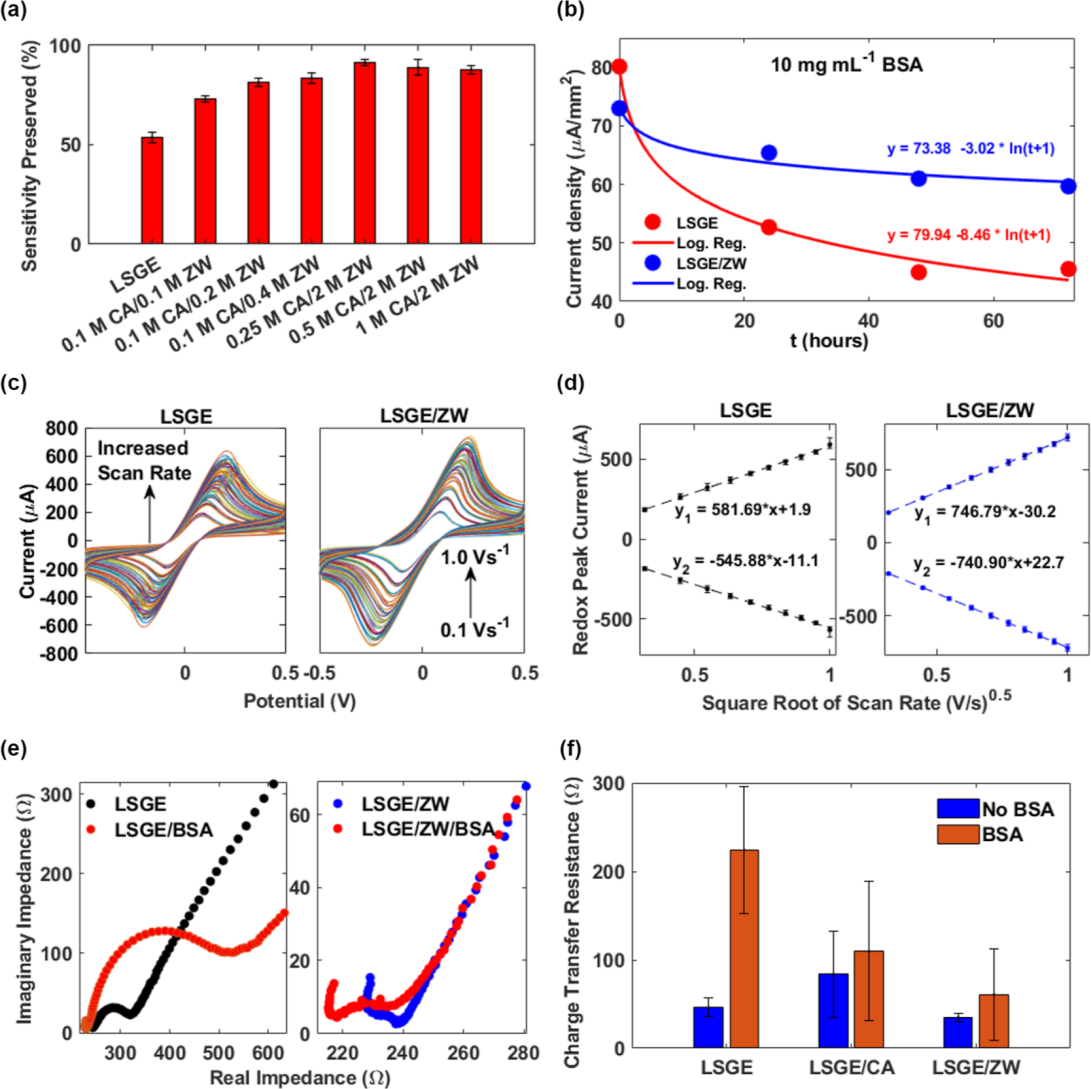
(a) Optimization and characterization of the LSGE/ZW interface with different coupling agent/ZW formulations (*n* = 4 independent electrodes). Error bars represent the standard deviations of the pooled samples. CA: carbodiimide/sulfosuccinimide coupling agent; BSA: bovine serum albumin. (b) Current density measurements in a period of 3 days of bare LSGE and LSGE/ZW immersed in 10 mg mL^–1^ albumin (*n* = 4 independent electrodes). (c) Cyclic voltammograms of bare LSGE and LSGE/ZW in 5.0 mM K_3_[Fe(CN)_6_]/K_4_[Fe(CN)_6_] in 0.1 M KCl at different scan rates (from 0.1 to 1.0 V s^–1^). (d) Reduction/oxidation peak current (*I*_p_) mean values plotted versus the square root of the scan rate of bare LSGE (*R*_LSGE(ox/red)_^2^ = 0.9990/0.9985) and LSGE/ZW (*R*_LSGE/ZW(ox/red)_^2^ = 0.9999/0.9999) (*n* = 6 independent electrodes). (e) Nyquist plots of bare LSGE and LSGE/ZW before and after immersion in 10 mg mL^–1^ albumin using 5.0 mM K_3_[Fe(CN)_6_]/K_4_[Fe(CN)_6_] in 0.1 M KCl. (f) Charge-transfer resistance bar plot of bare LSGE, LSGE/coupling agent, and LSGE/ZW before and after immersion in 10 mg mL^–1^ albumin solution (*n* = 3 independent electrodes).

Electrochemical measurements were performed to determine the optimal functionalization conditions required to minimize nonspecific adsorption of biomolecules and simultaneously maintain the sensor’s sensitivity. For this purpose, current density values of various LSGE/ZW settings, with different concentrations of coupling agent and ZW, were obtained from differential pulse voltammetry measurements, using the working electrode geometric surface area of 7.1 mm^2^ (Figure 2a). The electrochemical performance of the modified electrodes LSGE/ZW was evaluated using a potassium ferri-ferrocyanide redox probe {K_3_[Fe(CN)_6_]/K_4_[Fe(CN)_6_]}. The measurements were performed using albumin as an organic fouling agent, which is the most abundant protein in blood.^51^ By comparing the current density values of bare LSGE and modified LSGE/ZW before and after immersion in albumin solution, the original sensitivity of the electrodes as well as the percentage of sensitivity loss after biofouling adhesion can be evaluated. The optimized LSGE/ZW exceptionally preserved the current response after 24 h of immersion in a 10 mg mL^–1^ albumin solution, retaining up to 91.5% of the sensor’s sensitivity. A detailed explanation of the optimal parameters used in all of the experiments is presented in the Materials and Methods section.

Next, cyclic voltammetry measurements at different scan rates were taken for bare LSGE and modified LSGE/ZW to evaluate the influence of ZW functionalization on the electrochemical activity. The values obtained were used to calculate the electrochemically active surface area of the working electrode using the Randles–Sevcik equation (eq 1)
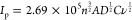
1

This equation expresses a linear correlation between the oxidation and reduction response currents (*I*_p_) and the square root of the scan rate (ν). Here, *A* denotes the electrode surface area (cm^2^), *C* represents the concentration of ferri-ferrocyanide (mol/cm^3^), n is the number of electrons involved in the redox event, ν is the scan rate (V/s), and *D* is the diffusion coefficient of the analyte (cm^2^/s). It was noted that the redox peak currents of the modified electrodes at each scan rate were higher than those of bare LSGE (Figure 2c,d). The electrochemically active surface area obtained for bare LSGE of 16.2 mm^2^ is comparable with the surface area found in the literature of 14 mm^2^ for LSGE fabricated using similar laser parameters and conditions.^52^ Taking into account that the geometrical surface area of the working electrode is 7.1 mm^2^, the real electrochemically active surface area corresponds to almost a twofold increase. This is primarily attributed to the porosity structure of LSGE and the multilayers formed in the ablation process. The modified LSGE/ZW electrodes showed an enhanced electrochemically active surface area of 21.4 mm^2^, corresponding to a 24.3% increase from that of the bare electrodes. On initial inspection, these results indicate that the coupling of ZW moieties increased the electrochemical activity of the electrodes. However, this could be attributed to the coupling agents’ activity which facilitates undesired side reactions. To determine if the ZW moieties were involved directly in the improvement of the electrochemical activity, the tests were repeated on a control sample treated with only the coupling agents (Figure S4). The electrochemically active surface area obtained for the coupling agents was 20.9 mm^2^, which is equivalent to a 22.4% increase from bare LSGE and comparable to the increase obtained from the ZW-functionalized samples. This suggests that the improvement can be at least in part attributed to the coupling agents. Although there is no direct evidence of carbodiimide/sulfosuccinimide being redox-active, we believe that their use has an important role as a surface modifier, responsible for the increase in the electrochemically active surface area.

Likewise, the charge-transfer resistance (*R*_ct_) of the ZW interface on LSGEs was assessed by electrochemical impedance spectroscopy. The impedance spectra of bare LSGE and modified LSGE/ZW were acquired using potassium ferri-ferrocyanide as a redox probe. Subsequently, the impedance spectra were fitted to the Randles circuit model. The circuit consists of a serial electrolyte resistance (*R*_s_) in series with a capacitor modeling the double-layer capacitance (*C*_dl_) that is in parallel with the *R*_ct_ and the Warburg diffusion element. Originally, bare LSGE presented *R*_ct_ values around 46.6 Ω, as depicted in Figure S6. Interestingly, the modified LSGE/ZW showed no significant variation in the *R*_ct_ with an average of 35.8 Ω (Figure S7). Therefore, the results suggest that the modification of the LSGE surface with ZW moieties does not increase the interfacial resistance and represents a low-impedance interface.

### Evaluation of the Antibiofouling Capacity

One of the biggest challenges for the use of point-of-care, wearable, and implantable devices is the nonspecific adsorption of proteins. These are adsorbed in nanoseconds on the surface of the materials, which adversely affects the performance of the device. Furthermore, when utilized in vivo, they initiate a foreign body response leading to a series of physiological events that result in inflammation of the surrounding tissue.^53,54^

Electrochemical measurements were carried out in order to evaluate how the performance of bare LSGEs is affected by protein biofouling. Furthermore, we verified whether the modification of the electrodes with ZW moieties provides an antibiofouling effect resistant to the nonspecific adsorption of proteins. As predicted, bare LSGE exhibited a significant loss of 46.3% in sensitivity after 24 h of immersion in 10 mg mL^–1^ albumin solution (Figure 2a). As the surface of the electrodes was rinsed with DI water before the measurements, the loss of sensitivity appears to be irreversible. On the other hand, as mentioned above, the ZW interface provides an outstanding fouling resistance effect against albumin preserving 91.5% of the sensor’s sensitivity.

Next, the stability of this ZW interface was analyzed at higher concentrations of albumin solution at room temperature. First, the current density values of LSGE and modified LSGE/ZW were recorded before and after 24 h of immersion in 10, 30, and 50 mg mL^–1^ of albumin solutions (Figure S8). After 24 h of immersion in 10, 30, and 50 mg mL^–1^ of albumin solution, the current response of bare LSGE decreased by ∼34.2, ∼34.4, and ∼32.4%, respectively. On the other hand, the current response of LSGE/ZW decreased ∼10.4, ∼11.5, and ∼14.7% for the three tested concentrations, respectively. The small variance in the reduction of the current responses at the different concentrations tested suggests that the concentration of albumin solution does not have a significant influence on the performance of the electrodes (Figure S9). Subsequently, the temporal stability of the performance was investigated by immersing the electrodes for 48 and 72 h using the same albumin concentrations (Figures 2b and S10). The current density response of bare LSGE experienced a drop of ∼43.9, ∼39, and ∼37.6%. In contrast, the current densities displayed by LSGE/ZW did not experience the same substantial decrease, suggesting that the ZW interface has maintained its performance over the testing period.

Additionally, a storage stability test was performed on LSGE/ZW for a period of 4 weeks. Over the span of the experiment, the electrodes were stored at 4 °C and sealed with parafilm to maintain controlled temperature conditions and avoid humidity. At the end of each week, the modified electrodes were immersed in 10 mg mL^–1^ albumin solution for 24 h, and the current density values were recorded. As can be seen in Figure S11, the current response of the electrodes is maintained for a minimum period of 4 weeks. This suggests that the ZW moieties are stable post-functionalization on the surface of LSGE and, as such, that the electrodes can be stored and used whenever needed.

To gain more insights into the antibiofouling effect, cyclic voltammetry measurements were collected after 24 h of immersion in 10 mg mL^–1^ albumin solution (Figure S5). These measurements gave us information about the electron-transfer kinetics between the analyte and the working electrode surface. For bare LSGE, the electrochemically active surface area was reduced by 23.8%, and a broader peak-to-peak separation was observed increasing from 0.4 V up to 0.5 V, indicating a limited diffusion of ferri-ferrocyanide due to biofouling. In contrast, modified LSGE/ZW exhibited a reduction in the real electrochemical surface area of 2.9% only. Additionally, the cyclic voltammetry “duck-shape” profile was conserved with a peak-to-peak separation of 0.4 V, indicating that the electrochemically active species had free access to the surface.

In the same manner, the impact of the fouling was assessed via electrochemical impedance spectroscopy after 24 h of immersion in 10 mg mL^–1^ albumin solution. The undesirable adhesion of proteins blocks the surface of the working electrode, which reduces the electrochemically active surface area. This manifests in an increase in the *R*_ct_ between the working electrode surface and the electroactive analyte. As expected, the *R*_ct_ of all electrodes tested increased after exposure to albumin, suggesting attachment of the proteins to the surface of the working electrode (Figure 2e,f). However, the magnitude of the increase is not equal for all the tested electrodes with the bare LSGE suffering from the largest increase in *R*_ct_ by 4.8 folds. In contrast, the LSGE/ZW samples had an increase of 1.73-fold in the *R*_ct_, which reflects the lower levels of protein adhesion to the surface.

The influence of the surface charge on the antibiofouling effect was also investigated by immersing the LSGE/ZWs in solutions containing myoglobin (pI = 7.0) and lysozymes (pI = 11) (Figure S14). At the testing conditions, the electrostatic repulsions between the biofouling agent and the ZW seem to play an important role in mediating biofouling resistance.

**Figure 3:**
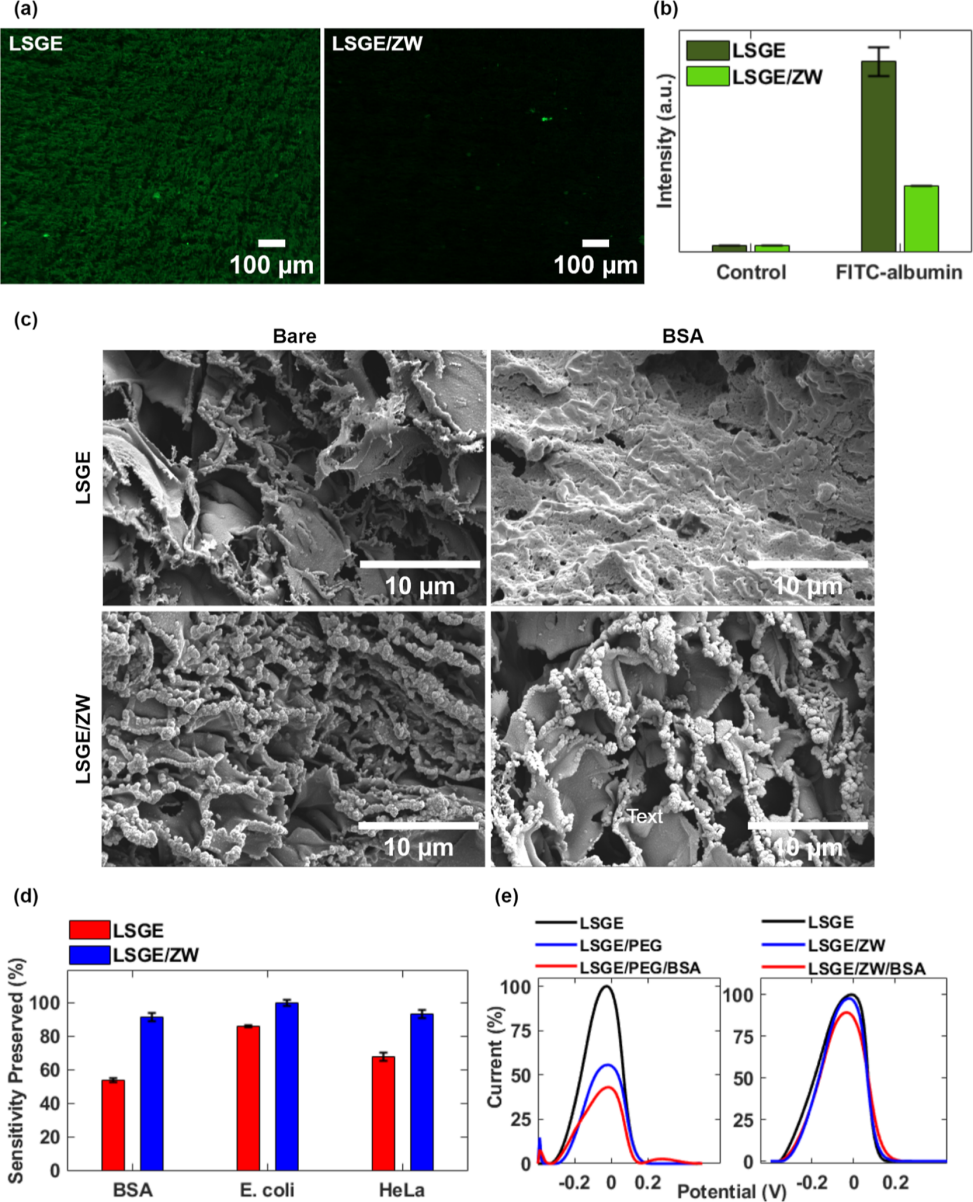
(a) Fluorescence microscopy images of bare LSGE and LSGE/ZW after 24 h of immersion in 10 mg mL^–1^ fluorescein-albumin solution. (b) Fluorescence intensity comparison between LSGE and LSGE/ZW before and after 24 h of immersion in 10 mg mL^–1^ fluorescein-albumin (FITC-albumin). (c) SEM images of bare LSGE and LSGE/ZW before and after immersion in 10 mg mL^–1^ albumin solution. BSA: bovine serum albumin. (d) Sensitivity preserved percentages of bare LSGE and LSGE/ZW after 24 h of albumin, *E. coli* bacteria, and HeLa cells incubation. (*n* = 4 independent electrodes). (e) Differential pulse voltammograms of LSGE/PEG and LSGE/ZW in a supporting electrolyte containing 5.0 mM K_3_[Fe(CN)_6_]/K_4_[Fe(CN)_6_] in 0.1 M KCl. Original peak current response (black) and functionalized electrodes (blue) after 24 h of immersion in 10 mg mL^–1^ albumin solution (red) (*n* = 4 independent electrodes).

The antibiofouling interface resistance to cell adhesion and biofilm formation on surfaces is also highly relevant for the development of biosensors.^22,29^ Applications such as implantable bioelectronics, wearable sensors, and in vivo biosensors require surfaces with antifouling properties against protein adsorption, cell growth, and bacterial attachments.^55,56^ In this work, we challenged the modified LSGE/ZW electrodes against the adhesion of HeLa cells and *E. coli* bacteria (Figure 3d). As with albumin, the electrochemical behavior was analyzed before and after 24 h of incubation via differential pulse voltammetry. Expectedly, the current density of bare LSGEs was reduced by 32 and 14% for cells and bacteria, respectively. Whereas modified LSGE/ZW reduced their sensitivity by 7 and 0.07% only, as can be seen in Figure 3d.

To better understand the antifouling effect exerted by the ZW moieties and corroborate our findings, the electrodes’ surface was investigated using microscopy techniques. First, fluorescence microscopy was utilized to visualize the distribution of fluorescein-albumin on the working electrode surface before and after 24 h of immersion. The fluorescence intensity in the bare LSGE was much higher than the intensity of the modified LSGE/ZW, as can be seen in Figure 3a. More specifically, the fluorescence intensity of the functionalized electrodes is approximately 80% less than that of the bare LSGE (Figure 3b).

Similarly, the surface of bare LSGE and modified LSGE/ZW was analyzed before and after immersion in 10 mg mL^–1^ albumin solution using SEM. The characteristic defects of graphene multilayers can be observed in the bare LSGE’s morphology with the presence of a highly wrinkled and porous surface, where the diameter of the pores ranges from 0.07 to 5.0 μm (mean = 3 μm, standard deviation = 1 μm) (Figure S12). Although these physical characteristics enhance the LSGEs’ performance by increasing the surface area and conductivity, they can also serve as sites for biomolecule adsorption. It is believed that once the biomolecules reach the surface, they can interact with the graphene structure through nonspecific interactions including van der Waals forces, π–π stacking, hydrogen bonding, hydrophobic interactions, and electrostatic forces.^53,57,58^ As can be seen in Figure 3c, adsorbed albumin spread and blocked the porous structure of LSGE, which explains the drastic loss of current density, reduction in the electrochemically active surface area, and increase in the *R*_ct_. Previously, Mücksch and Urbassek investigated albumin spreading behavior on a graphite surface and their observations corroborate our findings. The authors claim that, during the adsorption process, the protein suffers a conformational change where its lipid-binding domains bind the surface through hydrophobic interactions, causing its unfolding.^59^ It has been also reported that the basal plane of graphene promotes π–π stacking for the immobilization of proteins on the surface.^60^ On the other hand, the ZW-functionalized LSGE surface reveals a different picture, and the hydration layer and the electrostatic repulsion exerted by ZWs maintained the porous and wrinkled structure of the LSGEs by preventing the adsorption of albumin.

On the other hand, we observed that *E. coli* bacteria did not significantly block the pores of LSGE, as they were mainly positioned in the wrinkles of the graphene sheets (Figure S13). This particular distribution of *E. coli* is potentially due to their size, which can range from 1.0 to 2.0 μm in diameter. This is not sufficient to cover the majority of the graphene cavities. Furthermore, several studies have reported that laser-induced graphene electrodes have high resistance to biofilm formation because their morphology does not allow bacterial proliferation.^61^ This is in good agreement with the electrochemical tests presented previously where bacteria barely reduced the current density of bare LSGE. Nevertheless, the modified LSGE/ZW demonstrates superior antiadhesion properties against bacteria. A negligible number of bacteria embedded in the wrinkles of the functionalized LSGE was observed, with 99.9% of the sensitivity of the electrode being preserved after incubation. Contrary to bacteria, HeLa cell fragments were retained on the top of the graphene nanosheet edges, leading to a clear reduction in the porosity of the surface. As such, this is reflected in the significant reduction in the current density of the bare LSGEs. Moreover, the fetal bovine serum proteins present in the cell culture media have also contributed to this loss of sensitivity. In LSGE/ZW, no apparent HeLa cells, whole or fragments, were found to be attached to the surface. Consequently, 92.9% of the current response of the electrode was preserved.

### Assessment of an Alternative Antifouling Approach for LSGE

PEG is regarded as the antifouling gold standard for biomedical applications.^62^ As such, we investigated the relative performance of the ZW modification in relation to PEG. Our findings suggest that the introduction of PEG on the surface of LSGE has reduced the available surface area for electron transfer (Figure 3e). The modified LSGE/PEG electrodes exhibited a ∼44% reduction from the original LSGE current response after functionalization. This observation can be explained by the long chain length and insulating nature of PEG. Furthermore, it is consistent with previous findings, suggesting that PEG hinders the electron transfer between the porous surface of the LSGE and the redox species.^37^ In contrast, the integration of the ZW moieties on the LSGE does not appear to affect the current response. This is reflected in the limited 3.5% reduction of the current response of LSGE/ZW (Figure 3e). Therefore, although PEG is an effective antifouling agent for other applications, it would affect the response of the electrochemical sensors. ZW is a much better solution in this case.

In addition to PEG, there are other materials reported in the literature. For example, functionalization of glassy carbon with lubricin has been demonstrated to preserve 96% of the sensitivity after exposure to an albumin-containing solution.^63^ In the same vein, functionalization with a ZW-phenyl layer can also block the adhesion of biofouling agents.^40^ Alternative strategies have also been reported based on the patterning of the surface of the electrode.^64^ However, these strategies tend to increase the surface impedance and may not be suitable for porous electrodes such as LSGE. Interestingly, the presented ZW-based interface overcomes these limitations and compares favorably with what has been previously reported (Table S2).

## Conclusions

In summary, we modified the surface of porous LSGEs with an interface based on ZW moieties to make them resistant to the action of organics and biofouling. This interface provides the LSGE with a strong hydration layer and exerts electrostatic repulsion, which protects the electrode from various fouling agents. LSGE/ZW preserved the analytical performance and exhibited robust antifouling properties against albumin, *E. coli* bacteria, and HeLa cells, even after 24 h of exposure. Furthermore, the modified LSGE/ZW electrodes stored at 4 °C can be used for up to a month without significant loss of current density, and they allow several cycles of rinsing. The results of this study suggest that the LSGE/ZW is a promising alternative for developing implantable and point-of-care devices resistant to biofouling.

## Supporting Information Available

Characterization of the ZW composite, optimization of the ZW functionalization, influence of the protein charge in the antifouling performance, analysis of the electrochemically active surface area after albumin immersion, impedance study before and after functionalization and albumin immersion, investigation of the antifouling effect using higher concentrations of albumin solution, long-term extrapolation of the antifouling effect, storage stability test, porosity measurements of the electrodes, SEM images before and after cell and bacteria incubation, and comparison of the available antifouling strategies applied for carbon-based electrodes (PDF)
