# Supplementary material for: Porous Laser-Scribed Graphene Electrodes Modified with Zwitterionic Moieties: A Strategy for Antibiofouling and Low-Impedance Interfaces
Source: ACS Appl Mater Interfaces. 2024 Jan 17;16(4):4408–19. doi: 10.1021/acsami.3c15849 (PMC10835659; doi:10.1021/acsami.3c15849)
Supplement: Supplementary file 1 — am3c15849_si_001.pdf [file am3c15849_si_001.pdf]

SUPPLEMENTARY INFORMATION

Porous Laser-Scribed Graphene Electrodes Modified with Zwitterionic Moieties: A Strategy for Anti-Biofouling and Low-Impedance Interfaces

Alanis C. Zambrano [a,b,*], *Livia M. D. Loiola* [b,c], *Abdullah Bukhamsin* [a,b], *Radoslaw Gorecki* [b,c], *George Harrison* [d], *Veerappan Mani* [b,g], *Shadi Fatayer* [d,e], *Suzana P. Nunes* [b,c,f,*], *Khaled N. Salama* [a,b,g,*].

[a] Bioengineering Program, Biological and Environmental Sciences and Engineering Division, King Abdullah University of Science and Technology (KAUST), 23955-6900, Thuwal, Saudi Arabia

[b] Advanced Membranes and Porous Materials Center, King Abdullah University of Science and Technology (KAUST), 23955-6900, Thuwal, Saudi Arabia

[c] Environmental Science and Engineering Program, Biological and Environmental Sciences and Engineering Division, King Abdullah University of Science and Technology (KAUST), 23955-6900, Thuwal, Saudi Arabia

[d] Kaust Solar Center, King Abdullah University of Science and Technology (KAUST), 23955-6900, Thuwal, Saudi Arabia

[e] Applied Physics Program, Physical Science and Engineering Division, King Abdullah University of Science and Technology (KAUST), 23955-6900, Thuwal, Saudi Arabia

[f] Chemistry and Chemical Engineering Programs, Physical Science and Engineering Division, King Abdullah University of Science and Technology (KAUST), 23955-6900, Thuwal, Saudi Arabia

[g] Computer, Electrical and Mathematical Sciences and Engineering Division, King Abdullah University of Science and Technology (KAUST), 23955-6900, Thuwal, Saudi Arabia

Email: alanis.chicaizazambrano@kaust.edu.sa

ZW Characterization

ZW was characterized by FT-IR, ^{1}H NMR, and mass spectroscopy. As can be seen in **Figure S 1a**, the IR spectrum of the zwitterionic compound presents the O-H bond stretching broad band at around 3500 cm^{-1} related to water molecules adsorbed to this hydroscopic compound. The following bands are related to asymmetric and symmetric stretching modes of N-H and C-H bonds. C-H bond bending mode and also $C-N^+$ bond stretching mode are associated with weak bands observed at around 1400 cm^{-1}. The S=O stretching modes in the sulfonate group are associated with the strong bands observed at 1207 and 1151 cm^{-1}, while the S-O stretching mode is depicted by the band at 1028 cm^{-1}. The C-N stretching mode in primary aliphatic amines is normally observed in the 1250-1020 cm^{-1} range and therefore overlaps with sulfonate group vibrational modes. Finally, the low-intensity bands around 700 cm^{-1} are associated with aliphatic skeleton vibrations. C-S stretching mode band is expected to lower than 600 cm^{-1} and therefore is out of the acquired IR spectrum range.

Additionally, the ^{1}H NMR signals were analyzed and schematically associated to zwitterionic compound structure by the letters A to G: methyl groups ($-CH_3$) 6H_A at 1.255 ppm ($^3J_{AG}$ = 7.3 Hz), amino group ($-NH_2$) 2H_D at 2.680 ppm ($^3J_{DE}$ = 7.6 Hz), and methylene groups ($-CH_2-$) 2H_B at 1.896 ppm, 2H_C at 2.002 ppm ($^3J_{BC}$ = 7.5 Hz), 2H_E at 2.893 ppm ($^3J_{EH}$ = 7.5 Hz), 2H_F at 2.960 ppm ($^3J_{FC}$ = 7.5 Hz), 4H_G at 3.162 ppm and 2H_H at 3.241 ppm as depicted in **Figure S 1b**.

Finally, the mass spectrum of the compound 3-((2-aminoethyl)diethylammonium) propane-1-sulfonate (here referred to as ZW) is shown in **Figure S 1c**. The primary ion corresponding to the compound ZW is the peak at m/z 239.1428, which represents a protonated $[ZW+H]^+$ ion of the compound. As shown in the inset table in **Figure S 1c**, the calculated mass error is -1.6 ppm and

the topmost proposed formula by the Bruker Data Analysis Software matches that of the protonated species of ZW ($C_9H_{23}N_2O_3S$). The lower Sigma score for this formula shows that the observed ion has the closest isotopic pattern to the theoretical formula of protonated species of ZW among the possible formulas. The higher intensity signal at m/z 261.1247 corresponds to the sodiated species of ZW $[ZW+Na]^+$ (i.e. $C_9H_{22}N_2O_3SNa$). Signals at around m/z 117 correspond to the N,N-diethylethylenediamine starting amine respective positive ions, and higher m/z values signals correspond to ZW positive charged aggregates.

Optimization of the ZW functionalization

Two approaches were tested in order to optimize the concentrations of the solutions and obtain the highest antifouling effect. Initially, the concentration of the zwitterionic solution was varied using a geometric progression of (0, 0.1, 0.2, and 0.4M), while maintaining the carbodiimide:sulfosuccinimide solution constant at (0.1 M). However, the system was not fully saturated and the antifouling effect was not ideal. Therefore, the concentration of the carbodiimide:sulfosuccinimide solution was increased using a geometric progression of (0, 0.25, 0.5, and 1 M) with the zwitterionic solution set at a maximum of (2 M) in order to couple all the free carboxyl (-OOH) with the primary amine (-NH2) species.

Long-term extrapolation

The long-term behavior of LSGE and modified LSGE/ZW was extrapolated using logarithmic functions of time, with the form of $y(t) = a + c \times ln(t+1)$. These functions are fitted on the measurements of the current density for each group of electrodes and for each concentration of albumin solution as depicted in **Figure S10**. An F-test was used to analyze the goodness of the fit. As displayed in **Table S1b**, the coefficients of determination R^2 are close to 1, the F-values are

large, and the p-values are significantly less than the standard threshold 0.05, which indicates that this model has remarkable statistical significance. Therefore, our logarithmic assumption can be used reliably to estimate the current density values of the electrodes at future times. As can be seen in **Table S1a**, projections of the current density values after 30 days of albumin solution exposure of bare LSGE show a reduction in the current response of ~69.8%, ~64%, and ~64%, for 10 mg mL^{-1}, 30 mg mL^{-1}, and 50 mg mL^{-1}, respectively. In contrast, LSGE/ZW exhibits a reduction of ~27%, ~28%, and ~34%, for the different concentrations of albumin solution.

Investigation of the influence of surface charge on antifouling performance

To investigate the influence of protein charge on the ZW performance, LSGE and LSGE/ZW electrodes were immersed in both 30 mg mL^{-1} of myoglobin and lysozyme. The current response was measured before and after immersion to estimate the degree of biofouling after 24 hours. We observed that the LSGEs' current response was reduced by ~60% after immersion in the myoglobin solution, whereas LSGE/ZWs' response only decreased by ~28%. Myoglobin is composed of one short peptide chain with 153 residues and a heme group buried in a central hydrophobic pocket. The isoelectric point of myoglobin is at pH 7.0 and the immersion testing was conducted using 0.01 M PBS (pH 7.40) as the background. As such, the protein could be regarded as neutrally charged, thereby nullifying the effect of charge electrostatic repulsion and allowing it to adhere to the surface. Previously, at room temperature, myoglobin has been demonstrated to unfold at slightly basic pH levels, which releases the heme group to interact with the aqueous phase.[1] Consequently, this explains the higher reduction in the current response experienced by LSGE/ZW when immersed in myoglobin relative to that experienced when immersing in albumin. Contrastingly, the influence of electrostatic repulsion is highlighted in the lysozyme findings. The bare LSGEs and the LSGE/ZW experienced slight drops of ~8% and ~6%,

respectively, after immersion in a lysozyme-containing solution. As the aggregate charge of lysozymes is positive at the test pH of 7.4, we suspect that the larger degree of electrostatic repulsion played a role in preserving the current response in both electrodes. Furthermore, lysozymes have four native disulfide bonds and a number of interhelical interactions that stabilize the protein.[2,3] This stabilization mitigates unfolding at test conditions. This restricts the specific surface area of the protein which in turn limits the site of non-specific adhesion further reducing the potential for adherence of the protein onto the surface of the electrodes.

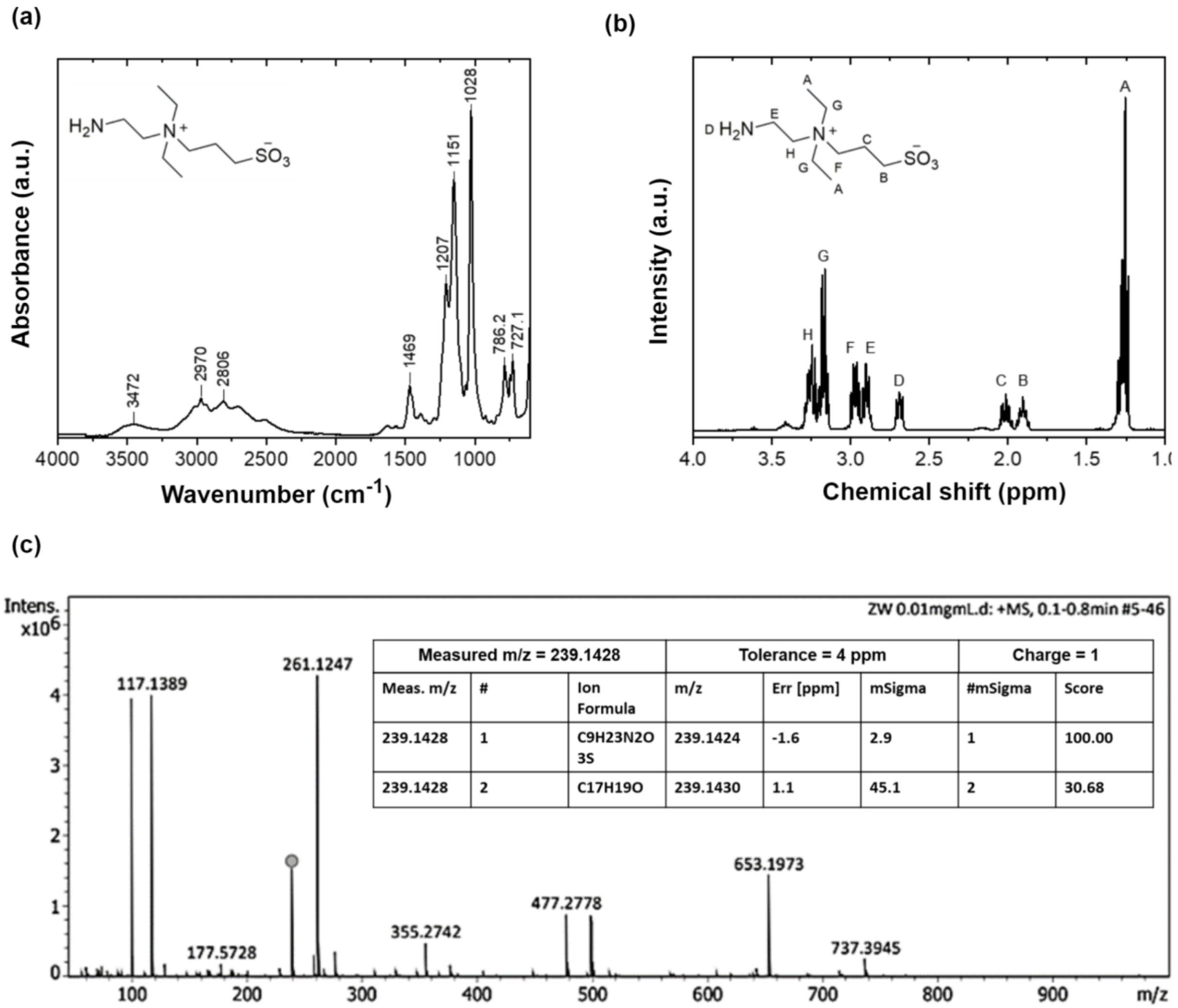

Figure S1. **(a)** FT-IR **(b)** ^{1}H NMR **(c)** mass spectroscopy of the ZW compound.

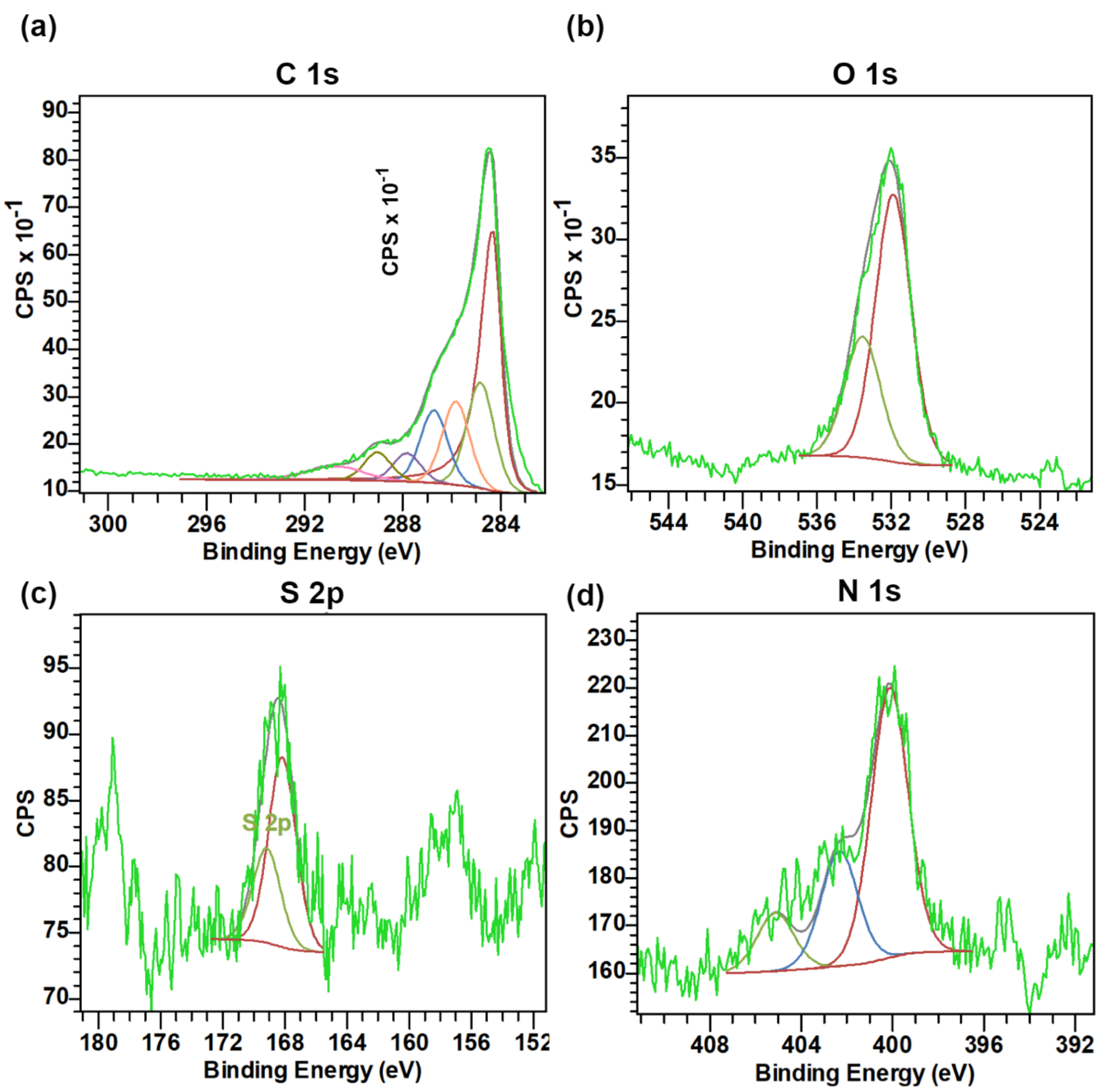

Figure S2. XPS **(a)** C 1s, **(b)** O 1s, **(c)** S 2p, and **(d)** N 1s deconvoluted spectra of LSGE/coupling agent.

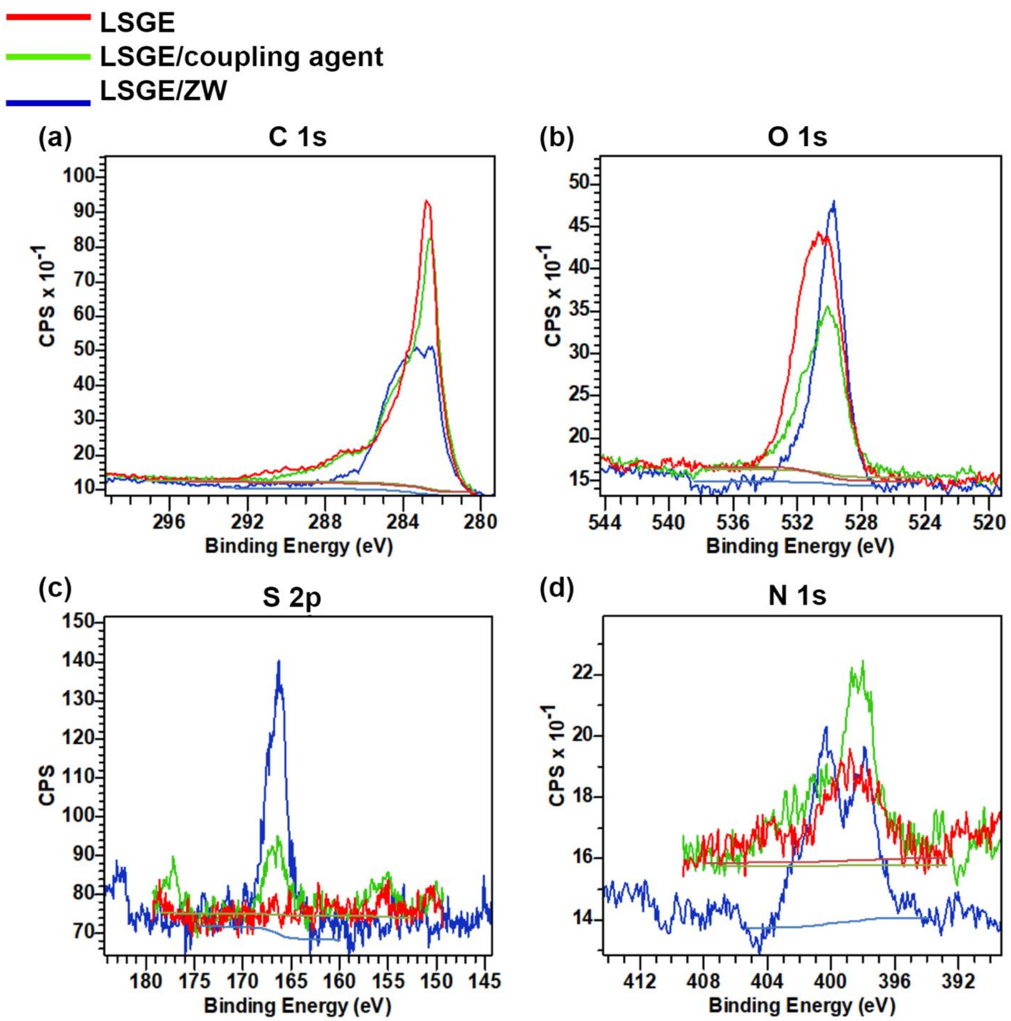

Figure S3. XPS **(a)** C 1s, **(b)** O 1s, **(c)** S 2p, and **(d)** N 1s spectra comparison between bare LSGE (red curve), LSGE/ZW (blue curve), and LSGE/coupling agent (green curve).

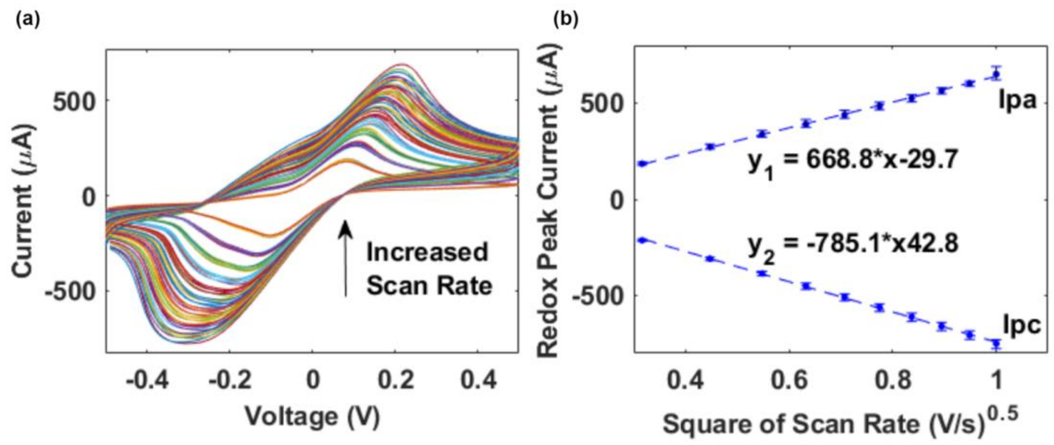

Figure S4. (a) Cyclic voltammograms of bare LSGE/coupling agent in 5.0 mM $K_3[Fe(CN)_6]/K_4[Fe(CN)_6]$ in 0.1 M KOH at different scan rates (from 0.1 V.s^{-1} to 1.0 V.s^{-1}). **(b)** Reduction/oxidation peak current (Ip) mean values plotted versus the square root of the scan rate ($R^2_{LSGE/coupling\ agent(ox/red)}$ = 0.9989/0.9993) (*n*= 6 independent electrodes).

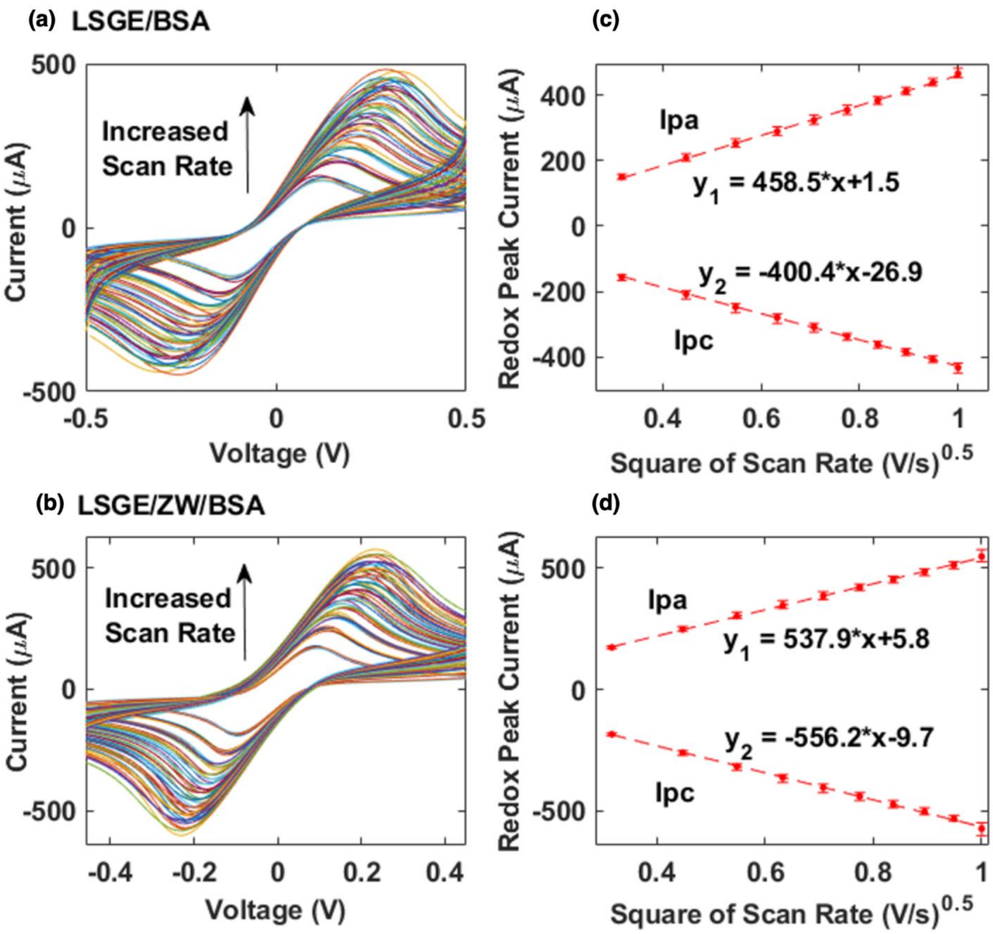

Figure S5. (a) CV voltammograms of bare LSGE (left) and **(b)** LSGE/ZW in 5.0 mM $K_3[Fe(CN)_6]/K_4[Fe(CN)_6]$ in 0.1 M KOH at different scan rates (from 0.1 $V.s^{-1}$ to 1.0 $V.s^{-1}$) after 24 hours immersion in a 1% albumin (BSA) solution. **(c)** Reduction/oxidation peak current (Ip) mean values plotted versus the square root of the scan rate of bare LSGE ($R^2_{LSGE/BSA\ (ox/red)}$ = 0.9994/0.9995) and **(d)** LSGE/ZW ($R^2_{LSGE/ZW/BSA\ (ox/red)}$ = 0.9996/0.9994) after 24 hours immersion in a 1% albumin solution (*n*= 6 independent electrodes).

(a)

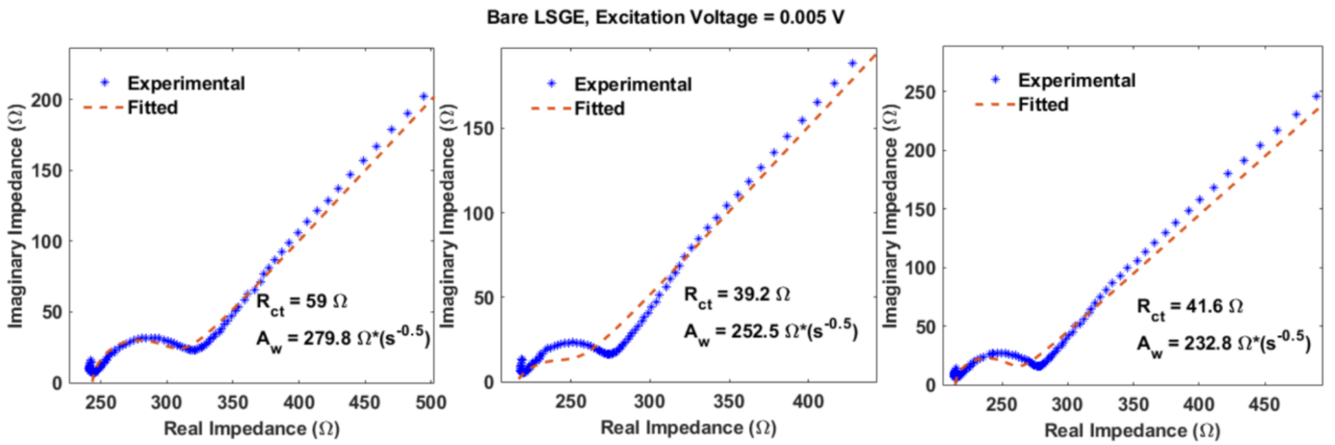

(b)

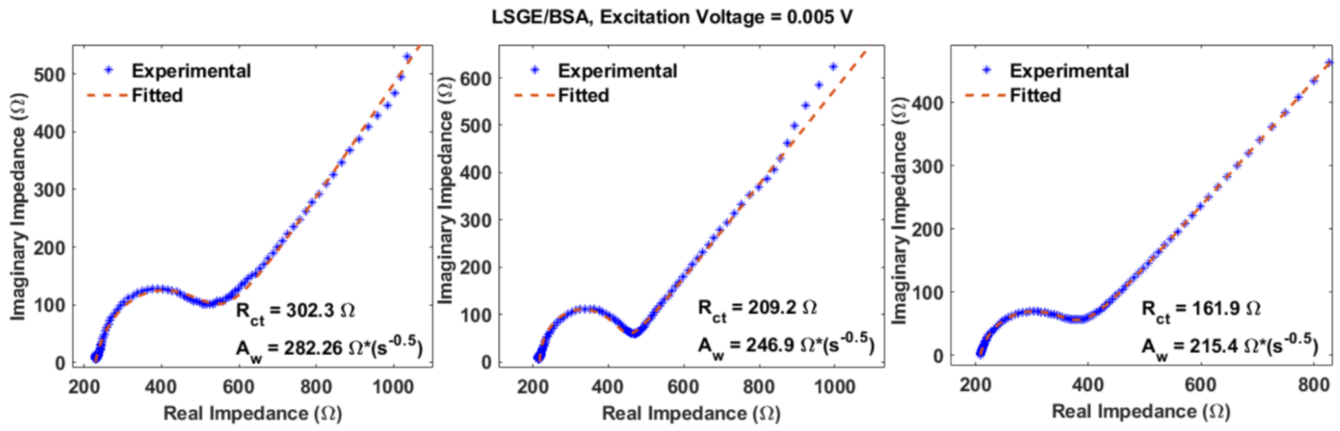

Figure S6. (a) Nyquist plots for bare LSGE electrodes prior to immersion in the albumin (BSA) solution. The Randles circuit model was used to plot an experimental fit (dashed line) and obtain values for the charge transfer resistance and the Warburg element (scribed in the plots). **(b)** Nyquist plots for the same electrodes in **(a)** after immersion in the albumin solution. EIS data of **(a)** and **(b)** are each performed in triplicate.

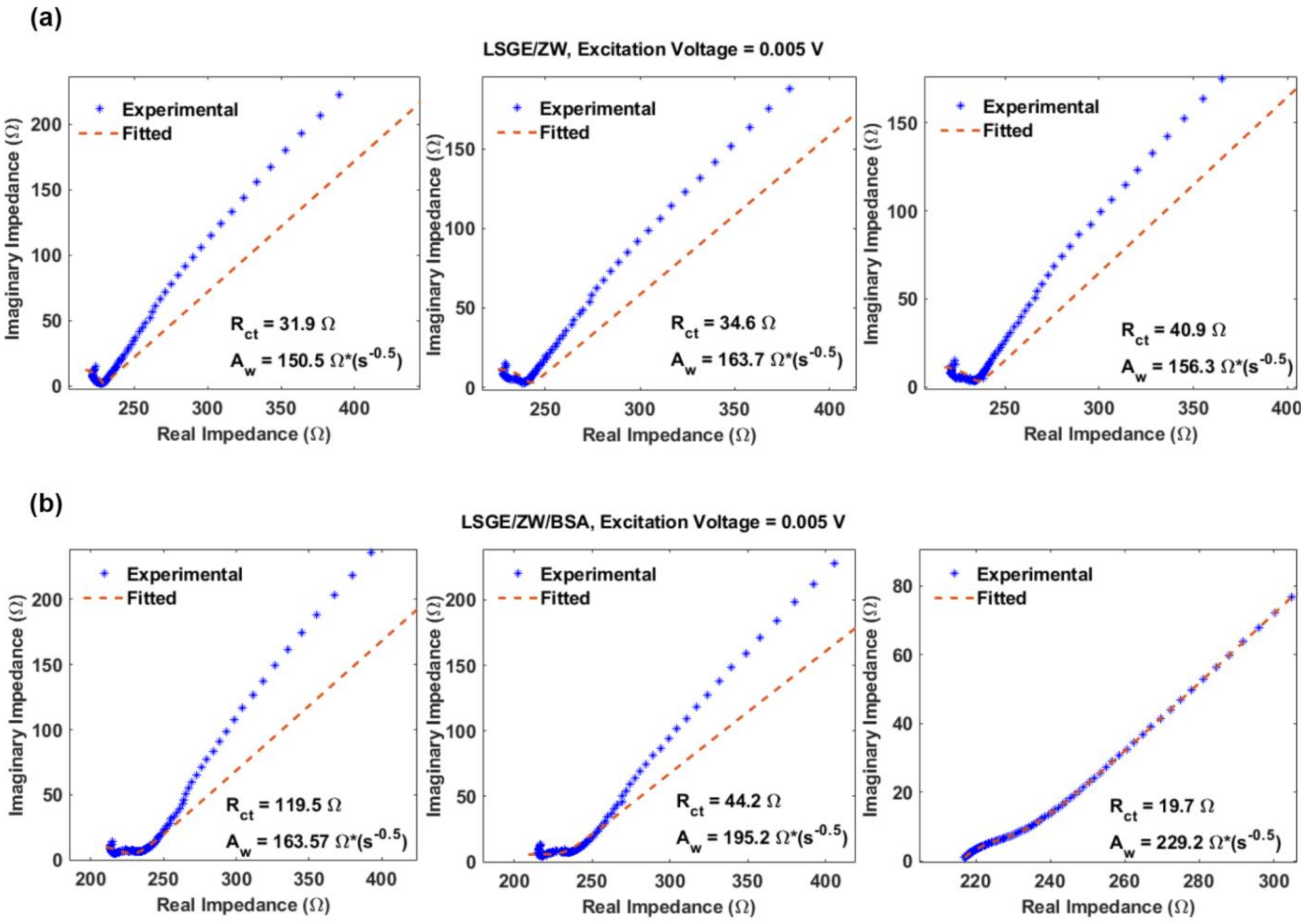

Figure S7. (a) Nyquist plots for functionalized LSGE/ZW electrodes prior to immersion in the albumin (BSA) solution. The Randles circuit model was used to plot an experimental fit (dashed line) and obtain values for the charge transfer resistance and the Warburg element (scribed in the plots). **(b)** Nyquist plots for the same electrodes in **(a)** after immersion in the albumin solution. EIS data of **(a)** and **(b)** are each performed in triplicate.

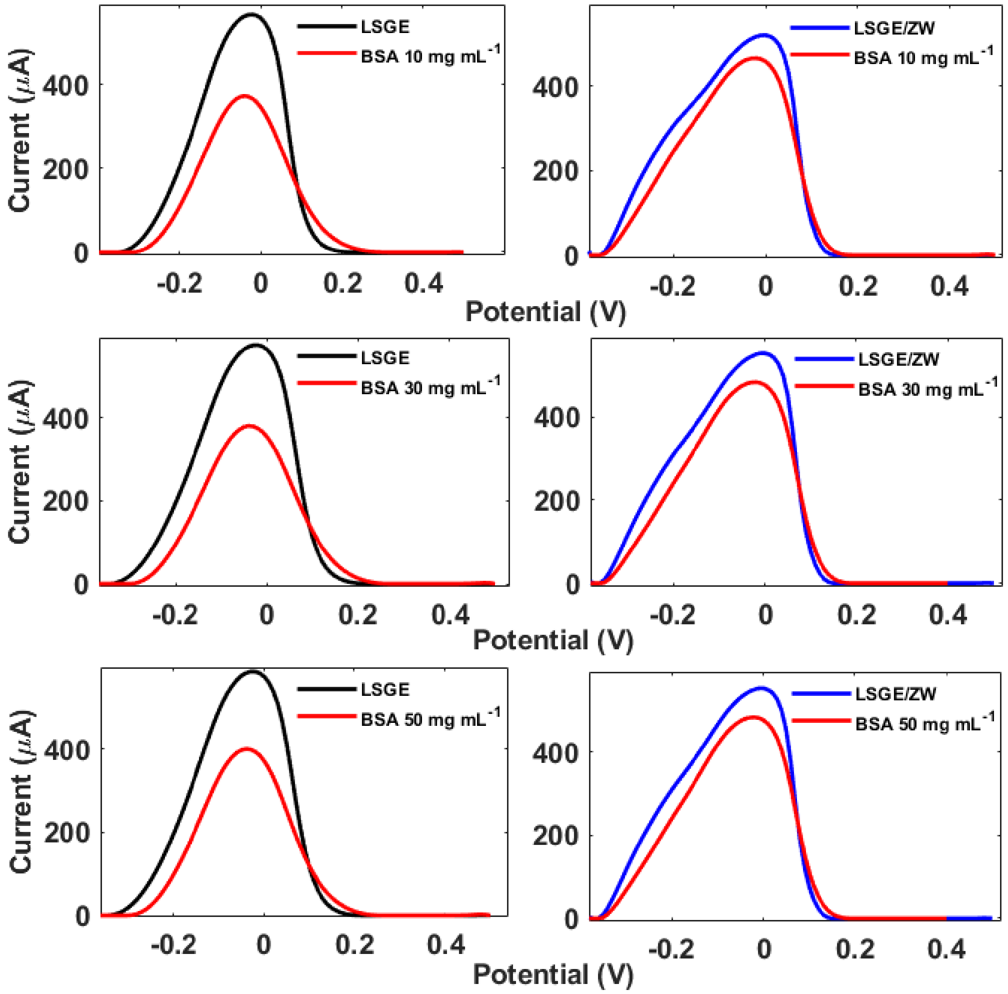

Figure S8. The current response of bare LSGE (black plot line, plot column on the left) and LSGE/ZW (blue plot line, plot column on the right) before and after (red plot lines) 24 hours of immersion in 10 mg mL^{-1} (top row), 30 mg mL^{-1} (middle row), and 50 mg mL^{-1} (bottom row) of albumin. The DPV test was performed using ferri/ferrocyanide as a redox probe.

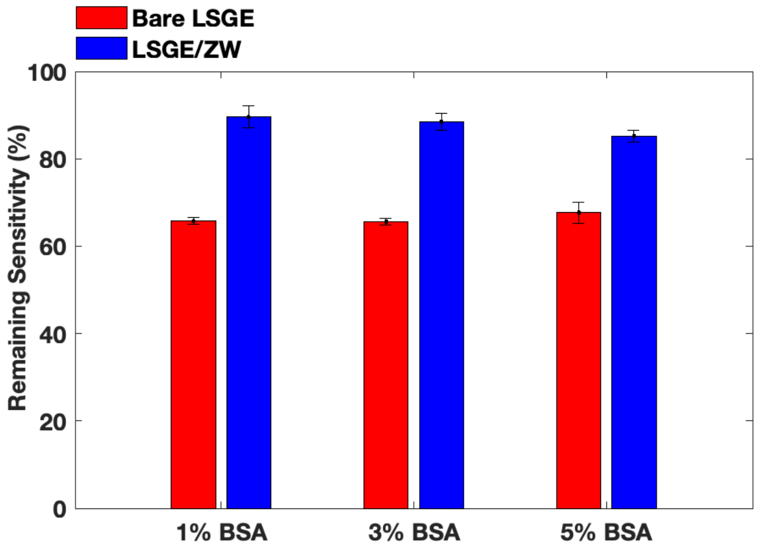

Figure S9. A bar plot of the calculated remaining current sensitivities before and after immersion in 10 mg mL^{-1}, 30 mg mL^{-1}, and 50 mg mL^{-1} of albumin for bare LSGE (red) and LSGE/ZW (blue). (n = 4 independent electrodes).

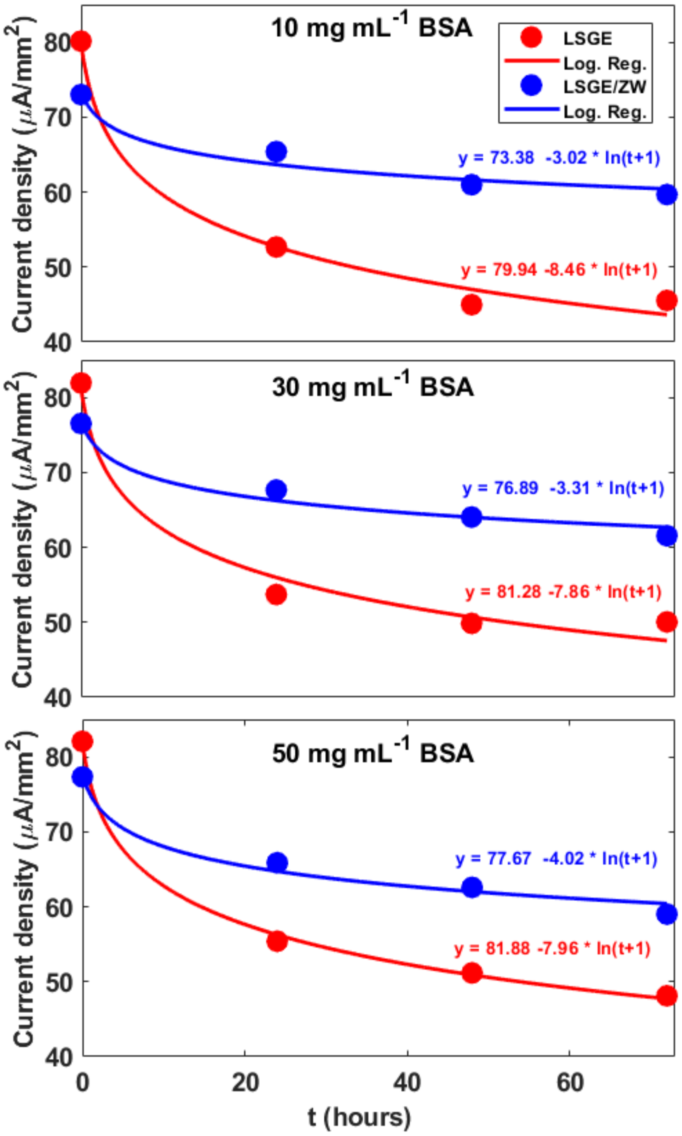

Figure S10. Current density measurements scatter-plotted alongside their logarithmic regressions, which is used to project their long-term behavior. The experimental data is approximated with the mathematical model with suitable accuracy.

Table S1: (a) Estimated current density values through logarithmic regressions (for short-term projection, the accuracy of the fit is adequate). **(b)** Statistical parameters related to each logarithmic regression.

(a)

	LSGE			LSGE/ZW		
Time \ BSA	10 mg mL^{-1}	30 mg mL^{-1}	50 mg mL^{-1}	10 mg mL^{-1}	30 mg mL^{-1}	50 mg mL^{-1}
0 h	79.9406	81.2770	81.8770	73.3827	76.8882	77.6658
24 h	52.7083	55.9696	56.2477	63.6509	66.2197	64.7141
48 h	47.0152	50.6803	50.8911	61.6163	63.9893	62.0064
72 h	43.6427	47.5470	47.7179	60.4111	62.6681	60.4024
730 h (1 month)	24.1513	29.4379	29.3785	53.4454	55.0320	51.1321

(b)

	LSGE			LSGE/ZW		
BSA \ Stat. Values	R^2	F	p-value	R^2	F	p-value
10 mg mL^{-1}	0.9905	209.5384	0.00059	0.9618	50.3795	0.00037
30 mg mL^{-1}	0.9826	113.1003	0.00091	0.9733	72.9597	0.00028
50 mg mL^{-1}	0.9986	1444.7351	7.2E-05	0.9803	99.6995	0.00030

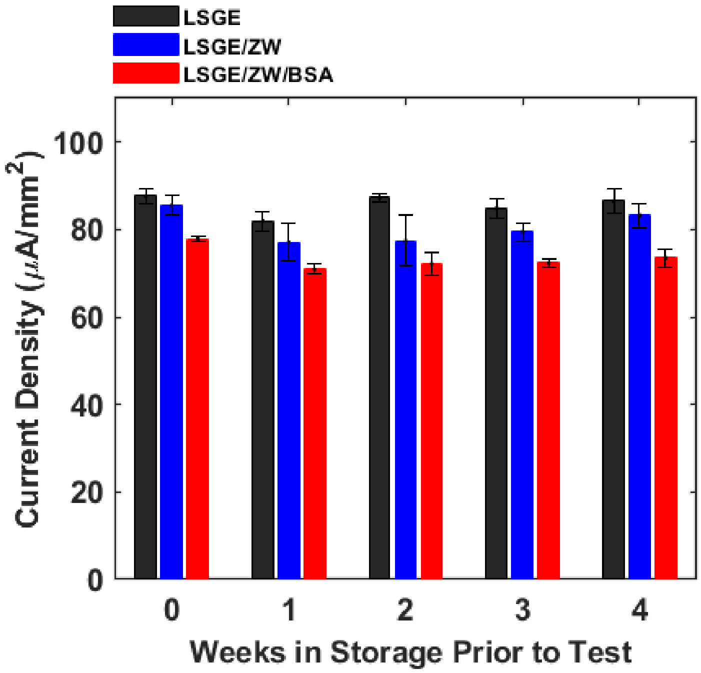

Figure S11. The current density recorded for bare LSGE (black), LSGE/ZW (blue), and LSGE/ZW after immersion in a solution containing albumin for 24 hours (red) and storage for several weeks. To conduct these tests, the electrodes were immersed in a solution containing ferri/ferrocyanide, which was utilized as a redox probe in a DPV test. (n = 3 independent electrodes)**.**

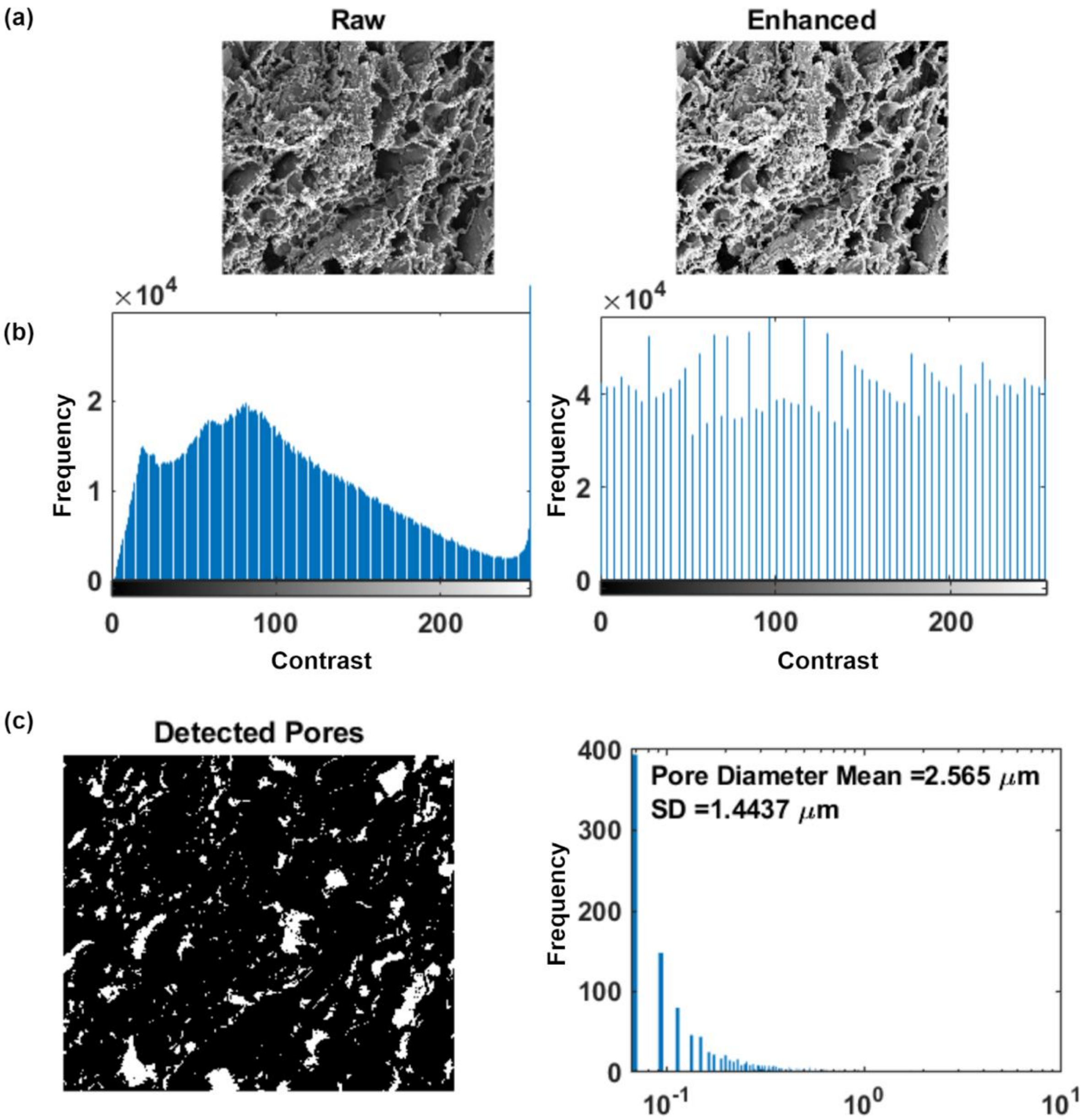

Figure S12. (a, b) Raw image obtained from the SEM contrast-enhanced to normalize the distribution of the binned pixels' intensity. (**c**) Enhanced image segmented to white and black regions based on a manually adjusted threshold where white indicates the presence of a pore. The size distribution of the pores was bar-plotted to estimate the dimensionality of the LSGE surface.

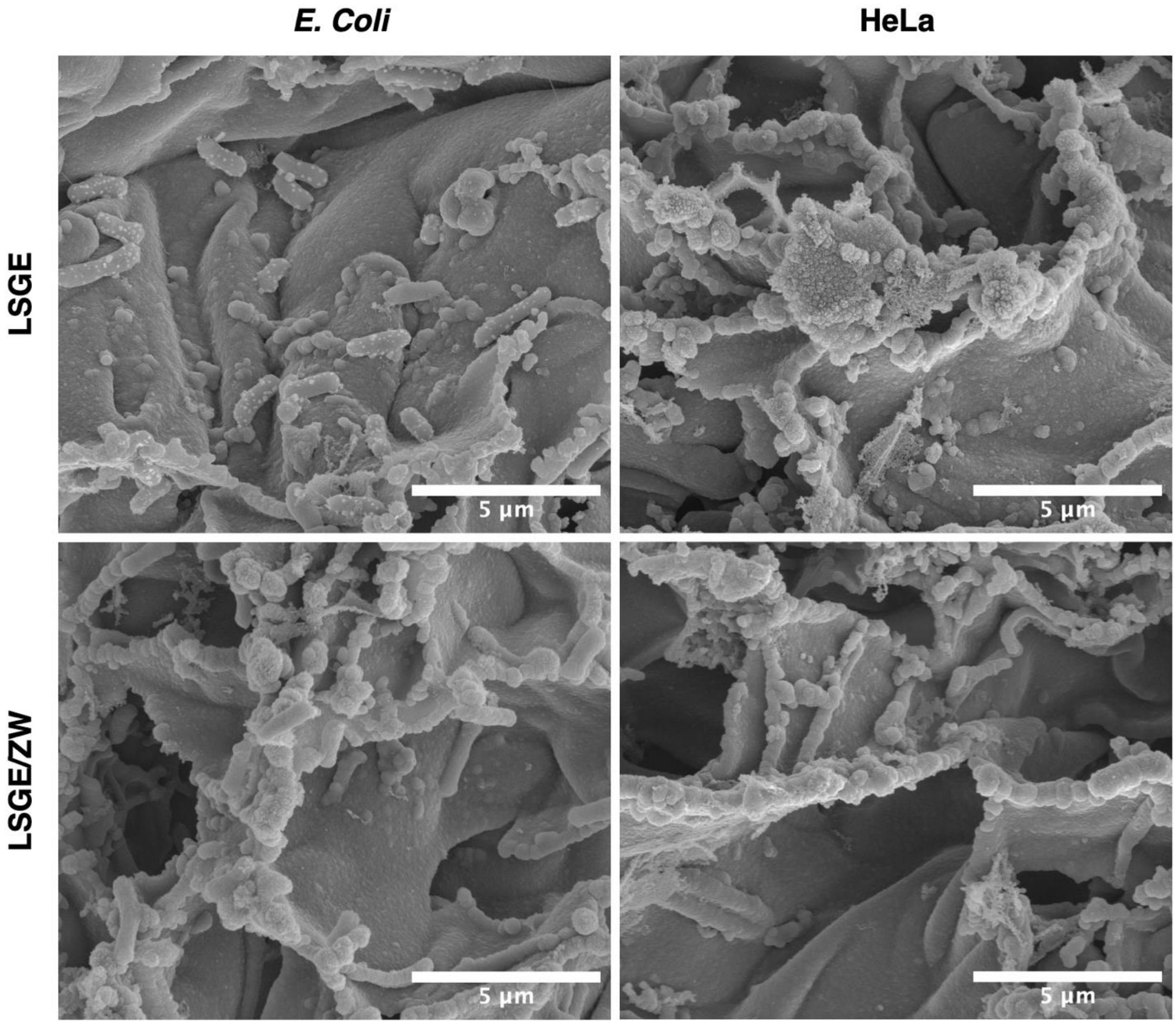

Figure S13. SEM images of bare LSGE and LSGE/ZW after *E. coli* bacteria and HeLa cells incubation.

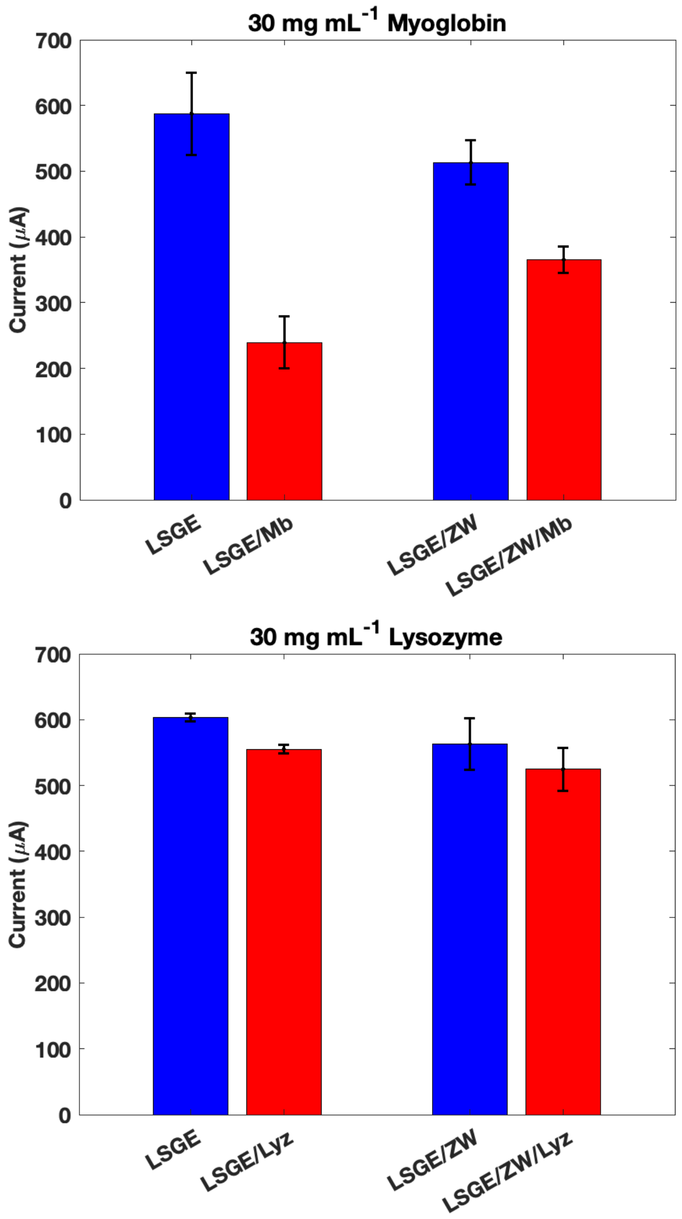

Figure S14. Current responses recorded for bare LSGE and LSGE/ZW before (blue) and after (red) immersion in solutions containing myoglobin (top) and lysozyme (bottom). To conduct these tests, the electrodes were immersed in a solution containing ferri/ferrocyanide, which was utilized as a redox probe in a DPV test. (n = 3 independent electrodes).

Table S2: Anti-biofouling strategies applied to carbon-based electrodes with performance benchmark comparisons.

Electrode	Antifouling Strategy	Sensitivity Loss (%) After Coating	Remaining Sensitivity (%) in Media	Ref.
LSGE	*Sulfobetaine-Zwitterions*	*3.5%*	*92.5% in BSA*	*Our work*
MWCNTs	Patterning electrode surface with nanostructures	N/A	94.1% in F12-K media	4
Graphite/SWCNTs	Polyethylene glycol	7.1%	N/A	5
Glassy carbon	Lubricin	Lowers the EASA by 15%	96% in 1% BSA	6
Glassy carbon	Zwitterionic Phenyl	Raises R_{ct} by 239 Ω cm^2)	R_{ct} changes by 7 Ω cm^2 after incubation with BSA	7
SPCE	Poly(N,N-dimethylacrylamide-stat-methacryloyloxy-benzo-phenone)/CNT	Polymer lowers the EASA by 70%	90% in BSA and Human Serum	8

References:

(1) Sykes, P.; Shiue, H.-C. S.; Walker, J.; Bateman, R. Determination of Myoglobin Stability by Visible Spectroscopy. *Journal of Chemical Education* **1999**, *76* (9), 1283–1284.
(2) Chang, J.-Y.; Li, L. The Unfolding Mechanism and the Disulfide Structures of Denatured Lysozyme. *FEBS Lett* **2002**, *511* (1–3), 73–78. https://doi.org/10.1016/s0014-5793(01)03284-7.
(3) Ghosh, A.; Brinda, K. V.; Vishveshwara, S. Dynamics of Lysozyme Structure Network: Probing the Process of Unfolding. *Biophysical Journal* **2007**, *92* (7), 2523–2535. https://doi.org/10.1529/biophysj.106.099903.
(4) Kousar, A.; Peltola, E.; Laurila, T. Nanostructured Geometries Strongly Affect Fouling of Carbon Electrodes. *ACS Omega* **2021**, *6* (40), 26391–26403. https://doi.org/10.1021/acsomega.1c03666.
(5) P.e., S.; Miller, T. S.; Meng, L.; Unwin, P. R.; Macpherson, J. V. Quantitative Trace Level Voltammetry in the Presence of Electrode Fouling Agents: Comparison of Single-Walled Carbon Nanotube Network Electrodes and Screen-Printed Carbon Electrodes. *Journal of Electroanalytical Chemistry* **2020**, *872*, 114137. https://doi.org/10.1016/j.jelechem.2020.114137.
(6) Russo, M. J.; Han, M.; Quigley, A. F.; Kapsa, R. M. I.; Moulton, S. E.; Doeven, E.; Guijt, R.; Silva, S. M.; Greene, G. W. Lubricin (PRG4) Reduces Fouling Susceptibility and Improves Sensitivity of Carbon-Based Electrodes. *Electrochimica Acta* **2020**, *333*, 135574. https://doi.org/10.1016/j.electacta.2019.135574.
(7) Gui, A. L.; Luais, E.; Peterson, J. R.; Gooding, J. J. Zwitterionic Phenyl Layers: Finally, Stable, Anti-Biofouling Coatings That Do Not Passivate Electrodes. *ACS Appl. Mater. Interfaces* **2013**, *5* (11), 4827–4835. https://doi.org/10.1021/am400519m.
(8) Zinggeler, M.; Schär, S.; Kurth, F. Printed Antifouling Electrodes for Biosensing Applications. *ACS Appl. Mater. Interfaces* **2022**, *14* (51), 56578–56584. https://doi.org/10.1021/acsami.2c17557.
